# Defining and Measuring Developmental Regression During Childhood: A Scoping Review

**DOI:** 10.1002/aur.70325

**Published:** 2026-08-08

**Authors:** Kirsten Furley, Jenny Fairthorne, Vishnu Priya Mohanakumar Sindhu, Katrina Williams, Amanda Brignell

**Affiliations:** ^1^ Department of Paediatrics Monash University Melbourne Victoria Australia; ^2^ Developmental Paediatrics Monash Children's Hospital Melbourne Victoria Australia; ^3^ School of Biomedical Sciences, Faculty of Health Queensland University of Technology Brisbane Queensland Australia; ^4^ Turner Institute for Brain and Mental Health, School of Psychological Sciences Monash University Melbourne Victoria Australia; ^5^ Department of Paediatrics Melbourne University Melbourne Victoria Australia; ^6^ School of Clinical Sciences Murdoch Children's Research Institute Melbourne Victoria Australia; ^7^ Department of Speech Pathology Australian Catholic University Melbourne Victoria Australia

**Keywords:** child development disorders, definition, developmental disabilities, developmental regression, neurodevelopmental disorders, pervasive

## Abstract

Developmental regression during childhood is inconsistently defined and measured, resulting in delayed diagnostic discovery and intervention. This study aimed to examine published definitions and measures for developmental regression. A comprehensive search strategy was applied to Medline, Embase, Cochrane, and PsycINFO databases. Reviewers independently screened relevant studies according to the *Preferred Reporting Items for Systematic reviews*. The number and percentages of definition use, their primary features, and measures were reported. Of 19,099 potential publications, 157 were included. Thematic analysis identified four condition groups: Neurodevelopmental disorders (*n* = 118); Progressive neurodegenerative conditions (*n* = 17); Developmental epileptic encephalopathies (*n* = 8); and Genetic conditions (*n* = 14). Most studies (*n* = 124, 79.0%) used an operational definition, and most specified types of skills lost (*n* = 147, 93.6%). Age of onset was specified in 67.5% (*n* = 106), and duration was specified in 34.4% (*n* = 54). Measures designed to specifically assess developmental regression were infrequently used (*n* = 5, 3.2%). We concluded that developmental regression is inconsistently defined and measured. A consistent approach is vital to ensure that all children are identified as early as possible. This will allow timely diagnostic discovery, enhance research rigor, and promote the collaboration that is needed to advance our understanding of causal mechanisms and effective interventions for affected children and their families.

**Abbreviations**
ADI‐R: Autism Diagnostic Interview‐Revised
CD: childhood dementia
CSWS
: continuous spikes and waves during sleep
DANR: Developmental and Neuro‐behavioral Regression Questionnaire
DEE: developmental epileptic encephalopathies
MLD: metachromatic leukodystrophy
PICO: population, intervention, comparison, outcomes framework
PIND: progressive intellectual and neurological deterioration
PMS: Phelan‐McDermid syndrome
PRISMA‐Sc: Preferred Reporting Items for Systematic reviews and Meta‐Analyses extension for scoping reviews
RSF: Regression Supplementation Form
RVI: Regression Validation Interview

## 1 Introduction

### 1.1 Background

Developmental regression is the loss of established skills during childhood. This regression may affect speech, impacting social and communication abilities. Other domains of loss include motor skills and functional skills such as toileting, feeding, or dressing. Developmental regression occurs in, but is not limited to, genetic or clinically classified neurodevelopmental disorders such as autism spectrum disorder (hereafter autism), developmental epileptic encephalopathies (DEE), and progressive neurodegenerative conditions. Although developmental regression is a recognized feature of each of these groups, not all children with these conditions experience developmental regression. Furthermore, a clear diagnosis of these overarching conditions is often not identified at the onset of the developmental regression.

### 1.2 Prevalence

As developmental regression occurs across a range of conditions, no single prevalence rate is available. Conditions causing developmental regression are often individually rare, albeit collectively not uncommon. For example, one paper (Djafar, Smith, et al. 2023; Djafar, Johnson, et al. 2023) reported an incidence of 0.036% for Childhood Dementia (CD), which is characterized by at least three months of progressive loss of previously acquired developmental or intellectual abilities and the presence of abnormal neurological signs. At the onset of regression, there may not be other signs or symptoms to point to a CD diagnosis.

Autism is the most frequently occurring neurodevelopmental condition that features developmental regression. Here, loss of skills is reported to have occurred in approximately 30% of children (Tan et al. 2021). In 2022, the Centers for Disease Control and Prevention (CDC) estimated the prevalence of autism in eight‐year‐olds across the US as 3.2%, an increase from 0.67% in 2000 (Shaw 2025). Therefore, based on the 2022 data, a minimum estimate of the prevalence of developmental regression is 1.0%.

### 1.3 Multiple Definitions of Developmental Regression

Researchers have used a variety of definitions for developmental regression during childhood and there are multiple possible explanations. First, conditions featuring developmental regression vary in their criteria. For example, the definition of CD excludes seizure disorders whereas these are included in the definition of Progressive Intellectual and Neurological Deterioration (PIND) if they feature progressive deterioration (Verity et al. 2021). Other discrepancies are the age of symptom onset, which is before 16 years for PIND but before 18 years for CD. Further, CD excludes cognitive deterioration when there are suspected acute metabolic crises, whereas this is not an exclusion for PIND (Verity et al. 2021). Second, neither the latest version of the Diagnostic and Statistical Manual of Mental Disorders, fifth edition, Text revision (DSM‐5‐TR) nor the latest version of the International Classification of Diseases 11th Revision (ICD‐11) include criteria for the diagnosis of developmental regression (American Psychiatric Association 2022; World Health Organisation 2019). As a result, when investigating or diagnosing developmental regression, researchers and clinicians have no standardized diagnostic criteria to reference, and this is likely to result in a range of definitions (Santoro et al. 2022). Third, the heterogeneity of conditions known to feature developmental regression may have contributed to the lack of an agreed definition. There are a range of such conditions and in each, the traits of the developmental regression vary considerably. In the context of autism, developmental regression is mentioned implicitly in DSM‐5‐TR where it states that for most, the onset of autism is associated with declines in critical social and communication skills, mostly occurring before 2 years (American Psychiatric Association 2022). Further and contrastingly, in Phelan–McDermid syndrome, regression usually affects motor and self‐help skills and occurs mostly after two and a half years (Reierson et al. 2017). A final example is Rett syndrome, where developmental regression is a core feature (Lee et al. 2013). The usual characteristics of developmental regression in Rett syndrome include an apparent stagnation of skills initially, followed by a rapid developmental regression occurring between 1 and 4 years of age. Areas of loss include fine and gross motor skills, language and social skills (Lee et al. 2013; Smeets et al. 2019). This diversity of presentation across disorders has likely hampered the development of a standardized definition of developmental regression.

Variable definitions have serious implications for research, the diagnosis of affected children, and services to families. For example, varying definitions create discrepancies between studies regarding eligibility criteria, thereby limiting comparisons between studies. Further, reported prevalence may differ between studies, with higher prevalence estimates resulting when broader definitions are used. This was demonstrated by a research group (Goin‐Kochel et al. 2014), which found that when the required duration of developmental regression was reduced from 3 months to 1 month, 11% more children were identified as having experienced developmental regression. This impacts the generalizability, clinical meaningfulness, and interpretation of research outcomes. Inconsistency creates the potential for slower identification and less reliable assessment, with potential delays to diagnosis, tailored treatment, care, and support for the child and family. Children with developmental regression may remain undiagnosed and instead remain only with a diagnosis of the overriding condition. This may be detrimental. For instance, children with autism and developmental regression usually have higher support needs and hence are more in need of services than those with autism and no regression (Reyes et al. 2024; Thompson et al. 2019). Consequently, the lack of formal criteria for the diagnosis of developmental regression means that funding for services is more difficult for parents to access. Finally, it is difficult for government policy in the area of disability to make provision for conditions which have no standardized definition.

In summary, the negatively impacted areas of imprecise definitions likely include: reducing clinically meaningful and translatable research outputs; limiting the generalizability and clinical impact of results; delayed diagnoses, treatment and support; an increased difficulty for families to access funding; and an inaccuracy in statistics to describe developmental regression. Overall, multiple definitions impede the growth of our knowledge and understanding of developmental regression. In light of the fact that developmental regression may no longer be considered a rare disorder and that the prevalence has continued to increase this century, it is crucial that steps are taken towards the development of a standardized definition.

### 1.4 Our Aims

We aimed to systematically search the literature to report the use of an operational definition of developmental regression and whether the primary features (onset, duration, and types of skills lost) were addressed. We also aimed to explore the more common primary and study‐specific measures used to measure developmental regression and to explore their use by condition group. Other aims were to examine the diversity of conditions featuring developmental regression and the number of publications exploring developmental regression over time. Achieving these aims will provide information to support the development of an agreed definition for developmental regression. In turn, this will make way for an improved understanding of developmental regression, along with more timely diagnoses and improved interventions, treatments, and support for families.

## 2 Methods

We completed the checklist from the *Preferred Reporting Items for Systematic reviews and Meta‐Analyses extension for scoping reviews* or PRISMA‐ScR (Tricco et al. 2018) and our protocol development followed this checklist, along with guidelines from the Joanna Briggs Institute (Aromataris et al. 2024).

### 2.1 The Search

Medline, Embase, Cochrane, and PsycINFO databases were searched. We used the *Population, Concept, and Context* method from the Joanna Briggs Institute (Aromataris et al. 2024) to frame the search strategy (Table 1).

**TABLE 1:** Search strategy applied to OVID Medline on 14 June 2025.

1.	Developmental disabilities/
2.	Neurodevelopmental disorders/
3.	Child Development Disorders, Pervasive/
4.	Autistic Disorder/
5.	1 or 2 or 3 or 4
6.	(regress* or deteriorat* or decline or loss or setback).ti,ab.
7.	(regress* adj1 (poisson or linear or univar* or multi* or adjust* or unadjust* or logis*)).ti,ab.
8.	(regress* adj1 (caudal or spinal)).ti,ab.
9.	5 and 6
10.	7 or 8
11.	9 not 10
12.	((neurocog* or intel* or cognitive) adj1 (regress* or decline* or deteriorat*)).ti,ab.
13.	((neurodevelopment* or autis*) adj1 (regress* or deteriorat* or decline or loss* or setback*)).ti,ab.
14.	((motor or psychomotor) adj1 (regress* or deteriorat* or decline or loss* or setback*)).ti,ab.
15.	((social* or language* or verbal or adaptive or behavior*) adj1 (regress* or deteriorat* or decline or loss* or setback*)).ti,ab.
16.	((skill* or milestone* or ability*) adj1 (loss* or decline or regress* or deteriorat*)).ti,ab.
17.	13 or 14 or 15 or 16
18.	12 or 13 or 14 or 15 or 16
19.	Limit 18 to “all child (0 to 18 years)”

*Note:* The syntax in the above table was used for Medline. Minor adjustments were made for its use with Embase, Cochrane, and PsycINFO.

Database searches were not limited by year of publication or language. For all but the most recent studies (those after June 2023), reference lists of included publications and forward citations were manually searched.

### 2.2 Eligibility and Exclusion Criteria

Studies were required to focus on developmental regression and participants were children aged from 0 to 18 years with loss in one or more of the developmental domains of speech, language, social, motor, cognitive, or adaptive skills. We excluded participants who presented with isolated motor loss related to a primary muscle disorder such as muscular dystrophy, those with reported toxin exposure, or acquired brain injury such as trauma or cerebral infection. Only peer‐reviewed research papers were eligible for inclusion, while reviews, meta‐analyses, and studies with fewer than 10 participants were excluded.

### 2.3 Data Extraction and Synthesis

Publications were imported into the Covidence Data Management System (Babineau 2014) and two reviewers screened at every stage from title and abstract (K.F., J.F., A.N., K.S., A.B., V.S., A.B., K.W.) to full text (K.F., J.F., A.N., K.S., A.B., V.S.). The first four mentioned persons extracted data, and data checking was completed by at least two reviewers. If required, conflicts were to be resolved by consultation with a third reviewer to reach a consensus.

An operational definition of developmental regression was defined as one that was clear and concise. For descriptions of conditions featuring developmental regression, we used thematic analysis, based upon the stages of Braun and Clarke (2006). The resulting condition groups were: *Neurodevelopmental disorders; Developmental epileptic encephalopathies; Neurodegenerative conditions*; and *Genetic conditions*, and each study was placed into exactly one condition group. Types of skills lost were categorized as: *Language*; *Motor*; *Cognitive*; *Language and motor*; *Language and social*; and *Mixed*. The last category was used when more than one of the former categories was involved and not covered by either *Language and motor* or *Language and social*.

By study and condition group, we report the number and percentage of studies using:

1. An operational definition;
2. Age of onset, duration, and types of skills lost as fundamental features of developmental regression;
3. Standardized measures to assess skill loss and the features of the more common of these measures; and
4. Study specific and non‐standardized measures to assess skill loss.

We also report the specific conditions which were the focus of included studies and the frequency of studies by time of publication.

## 3 Results

### 3.1 Included Studies

**FIGURE 1:**
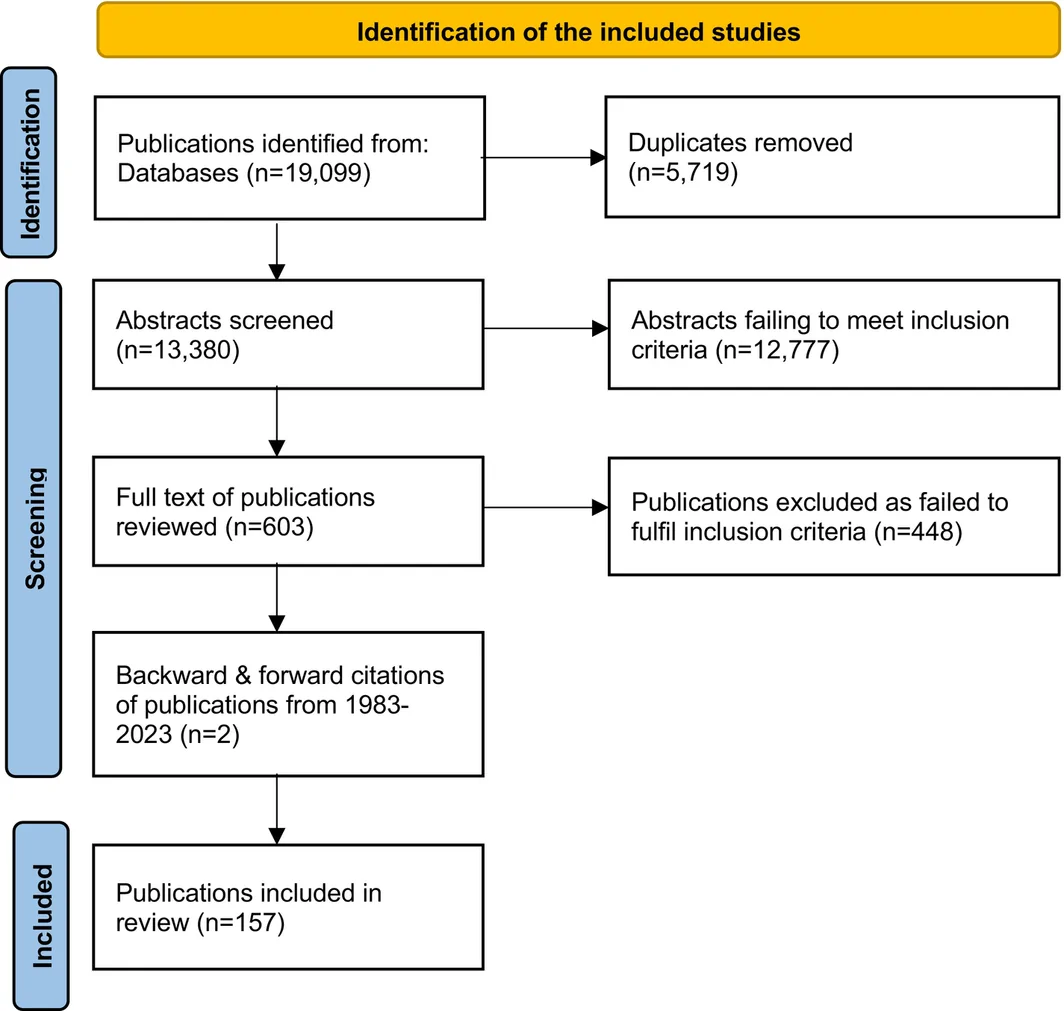
Identification of the included studies. Proforma for flowchart obtained from the PRISMA Webpage (Tricco et al. 2018).

In total, 19,099 publications were identified from 1963 to 2025 and 5719 duplicates were removed (Figure 1). Of the remaining 13,380 publications, 12,777 failed to meet our inclusion criteria at the initial screening of abstracts and were excluded. The remaining 603 publications required full‐text review, and 448 of these were excluded as either the full text was unavailable, they were not peer‐reviewed research, or they did not fulfill our other eligibility criteria. Two were added after checking forward and backward citations of included papers published in or before June 2023, leaving 157 publications for the final analyses (Figure 1). None of the decisions required a third reviewer to resolve conflicts. Included studies are listed in Table S1.

#### 3.1.1 Operational Definition

Most studies, (*n* = 124, 79.0%) included an operational definition for developmental regression (Table S1). Studies focusing on neurodevelopmental disorders used an operational definition most often (*n* = 98, 83.1%) while studies exploring DEE (*n* = 56, 2.5%) had the lowest use. More than 60% of studies focusing on progressive neurodegenerative conditions (*n* = 11, 64.7%) and more than 70% of studies focusing on genetic conditions (*n* = 10, 71.4%) used an operational definition (Table S1). Notably and as indicated by the variation in reported features of developmental regression, the operational definition varied between studies (Table S1).

### 3.2 Features of Developmental Regression

Data pertaining to the features of the regression are given, by study, in Table S1, and are summarized below.

#### 3.2.1 Age at Onset

More than two‐thirds of publications (*n* = 106, 67.5%), included the age of regression onset (Table S1). Studies of neurodevelopmental disorders had the highest inclusion rate of age of onset (*n* = 98, 83.1%) whilst only 62.5% (*n* = 10) of DEE studies included this information. Genetic conditions provided age of onset in 10 studies (71.4%) while 11 studies (64.7%) of progressive neurodegenerative conditions provided the age of onset.

#### 3.2.2 Duration of Regression

Only 34.4% (*n* = 54) of publications specified the duration of regression. Studies focusing on progressive neurodegenerative conditions were the most likely to include the duration of regression (*n* = 8, 47.1%), and DEE studies were the least likely (*n* = 1, 12.5%). Of neurodevelopmental studies, more than a third described the duration of regression (*n* = 41, 34.7%), and of studies of genetic conditions, four (28.6%) specified the duration of the regression.

#### 3.2.3 Types of Skills Lost

Most studies (*n* = 147, 93.6%) specified the types of skills lost in relation to developmental regression. The most described area of loss was the *Mixed* category (*n* = 87, 55.4%) and the least described was the *Language and Motor* (*n* = 1, 0.6%) and *Motor* categories (*n* = 1, 0.6%). In descending order of percentage, the frequencies and percentages of other categories were: *Language and social* (*n* = 32, 20.4%); *Language* (*n* = 22, 14.0%); *Not described* (*n* = 10, 6.4%); and *Cognitive* (*n* = 4, 2.5%).

The proportion not specifying types of skills lost was lowest for studies of progressive neurodegenerative conditions (*n* = 0, 0%) and highest for studies of DEE where two studies (25.0%) did not specify the types of skills lost (Table 2). Seven (5.9%) studies of neurodevelopmental disorders, two studies (25%) of DEE and one study of genetic conditions (7.1%) did not specify the types of skills lost.

**TABLE 2:** Area of skill loss by condition group.

Area of loss	Condition group				Total
	Neurodevelopmental disorders	Progressive neurodegenerative conditions	Developmental epileptic encephalopathies	Genetic conditions	
Cognitive	1 (0.8%)	0 (0%)	3 (37.5%)	0 (0%)	4 (2.5%)
Language	18 (15.3%)	0 (0%)	3 (37.5%)	1 (7.1%)	22 (14.0%)
Language and motor	0 (0%)	0 (0%)	0 (0%)	1 (7.1%)	1 (0.6%)
Language and social	32 (27.1%)	0 (0%)	0 (0%)	0 (0%)	32 (20.4%)
Mixed	60 (50.8%)	16 (94.1%)	0 (0%)	11 (78.6%)	87 (55.4%)
Motor	0 (0%)	1 (5.9%)	0 (0%)	0 (0%)	1 (0.6%)
Not described	7 (5.9%)	0 (0%)	2 (25.0%)	1 (7.1%)	10 (6.4%)
Total	118 (100%)	17 (100%)	8 (100%)	14 (100%)	157 (100%)

By condition group, the percentage of studies describing loss in different areas varied considerably. For example, for neurodevelopmental disorders, 50.8% (*n* = 60) of studies described loss in the *Mixed* category, 27.1% (*n* = 32) in the *Language and social* category, and 15.3% (*n* = 18) in the *Language* category. Contrastingly, for genetic conditions, 78.6% (*n* = 11) of studies described skills lost as in the *Mixed* category and 7.1% (*n* = 1) of studies in each of the *Language*, *Language and motor*, and *Not described* categories (Table 2).

### 3.3 Measures of Regression and Their Use by Condition Group

#### 3.3.1 Standardized Measures

Across studies, the ADI‐R was the most used standardized measure of regression (*n* = 53, 33.8%). Fifty‐two (44.1%) neurodevelopmental studies used the ADI‐R with the only other use being by a genetic study (7.1%). In most of these studies (*n* = 51, 96.2%), the ADI‐R was used with other standardized measures. For example, in 33 (62.2%) studies, the ADI‐R was used in conjunction with the *Autism Diagnostic Observation Schedule*, in three (5.8%) with the *Regression Supplementation Form* (RSF) and in one with the Regression Validation Interview (RVI). Another regression specific measure, the *Developmental and Neurobehavioral Regression Questionnaire* (DANR) was used in only two (1.3%) studies of a neurodevelopmental condition (Table 3).

**TABLE 3:** By paper and condition group, key measures used to assess developmental regression.

Key measure(s) used	Condition group				
	Neuro‐developmental conditions	Progressive neurodegenerative conditions	Developmental epileptic encephalopathies	Genetic conditions	Total
ADI‐R	2	0	0	0	2
ADI‐R and other	38	0	0	1	39
ADI‐R and video	1	0	0	0	1
ADI‐R, DANR and other	1	0	0	0	1
ADI‐R, RVI and other	1	0	0	0	1
ADI‐R, video and other	4	0	0	0	4
ADI‐R, video, RSF and other	2	0	0	0	2
DANR and other tools	1	0	0	0	1
Not described	6	2	0	0	8
Other tools	43	9	7	8	67
Study specific measure only	6	2	0	1	9
Study specific measure and other	7	3	0	1	11
Study specific measure and video	1	1	0	0	2
Study specific measure, ADI‐R and other	2	0	0	0	2
Study specific measure, ADI‐R, RSF and other	1	0	0	0	1
Video and other tools	2	0	1	2	5
Video only	0	0	0	1	1
Total	118 (100%)	17 (100%)	8 (100%)	14 (100%)	157 (100%)

*Note: Key measure* refers to the primary methods used to collect data.

Abbreviations: ADI‐R, Autism Diagnostic Interview‐Revised; DANR, Developmental and Neuro‐behavioral Regression Questionnaire; RSF, Regression Supplementation Form; RVI, Regression Validation Interview.

Overall, there were four measures used to specifically assess loss of skills. These were the ADI‐R, the DANR, the RVI and the RSF. Two (ADI‐R and RSF) enquired about social and language loss, with options for asking about loss in other developmental domains. The DANR enquired about social, language, gross and fine motor loss while the RVI enquired about social and language, and socio‐communication losses. All four measures enabled respondents to describe skill use prior to the regression. There was variation between measures with respect to the duration of the loss with the ADI‐R and the RVI enquiring only of losses of at least 3 months and 1 month respectively. On the other hand, the RSF and DANR had no minimum duration. Three measures allowed the respondent to give any age of onset of the loss while the ADI‐R specified a maximum allowable age of 36 months for the onset. Only the DANR allowed the respondent to describe events concurrent with the onset of the regression (Table 4).

**TABLE 4:** Standardized measures to assess regression and their primary features.

Measure	Use by condition group	No. of studies	Regression features of measure				
			Type of skill loss	Prior skill use	Duration of loss	Age of onset	Concurrent events
ADI‐R (Kim et al. 2013)	Neuro‐developmental and genetic	52 ND, Genetic 1	Asks of social and language with options for other domains	Yes, daily use	Yes, ≥ 3 months	Yes, before 36 months	Not specified
RSF (Goldberg et al. 2003)	Neuro‐developmental	3	Asks of social and language with options for other domains	Yes	Yes	Enquired	Not specified
DANR (Frye et al. 2020)	Neuro‐developmental	2	Asks of social, language, gross and fine motor	Yes	Yes	Enquired	Enquired
RVI (Thurm et al. 2014)	Neuro‐developmental	1	Asks of social and language, and socio‐communication	Yes	Yes, ≥ 1 month	Enquired	Not specified

Abbreviations: ≥, at least; ADI‐R, Autism Diagnostic Interview‐Revised; DANR, Developmental and Neurobehavioral Regression Questionnaire; DEE, Developmental epileptic encephalopathies; ND, Neurodevelopmental; RSF, Regression Supplementation Form; RVI, Regression Validation Interview.

#### 3.3.2 Study Specific Measures

Of the 149 (94.9%) studies using a key measure, 25 (16.8%) used a study specific measure where at least one of the research team had been involved in its development (Table 3). There were differences in the use of such measures by condition group (Table 3). Proportionately, studies of progressive neurodegenerative conditions used study specific measures most often (35.3%, *n* = 6), studies of neurodevelopmental disorders and studies of genetic conditions had approximately equal use (15.2%, *n* = 17 and 14.2%, *n* = 2, respectively), while no studies of DEE used a study specific measure (Table 3).

Of the 25 papers using a study specific measure, nine (36%) used these with no other measures of regression, while two (8%) used a study specific measure in conjunction with videos. Three (12%) papers used a study specific measure, along with the ADI‐R and other measures, with the remaining eleven (44%) using a study specific measure in conjunction with measures which did not include the ADI‐R (Table 3).

#### 3.3.3 Other Non‐Standardized Measures

Videos were used, with and without other measures in 15 studies (9.6%) with most of these being studies of neurodevelopmental disorders (*n* = 10, 71.4%). Three studies of genetic conditions (21.4%), one study of progressive neurodegenerative conditions (5.9%), and one study of DEE (7.1%) also employed videos to assess regression. Each of these 15 studies used a research‐specific approach to code the home videos (Table 3).

Other non‐standardized measures used to assess a regression were clinical assessment, medical records, parent information, school records, and various combinations of these measures. Of these measures, the most frequently used were medical records, which were used either as the only non‐standardized measure (*n* = 18, 11.5%) or in conjunction with other non‐standardized measures (*n* = 25, 15.9%). Clinical assessment was the second most frequently used of these measures. Again, clinical assessment was sometimes the only reported non‐standardized measure to assess the regression (*n* = 8, 5.1%) whilst at other times it was used in conjunction with other non‐standardized measures (*n* = 18, 11.5%).

For studies of neurodevelopmental disorders, the most used measures were parent information (*n* = 26, 22.0%) and medical records (*n* = 15, 12.7%). For studies of progressive neurodegenerative conditions, the most used measures were medical records (*n* = 3, 17.6%) and parent information (*n* = 2, 11.8%). In studies of DEE, three (37.5%) gained information about regressions from medical records and one (12.5%) from parent information. For studies of genetic conditions, medical records were used in three (21.4%) studies and parent information in one (7.1%) study. See Table 5.

**TABLE 5:** By condition group, non‐standardized regression measures assessing developmental regression by number and percentage.

Non‐standardized measure(s)	Condition group				
	Neuro‐developmental disorders	Progressive neurodegenerative conditions	Developmental epileptic encephalopathies	Genetic conditions	Total
Clinical assessment only	5 (4.2%)	1 (5.9%)	0 (0%)	2 (14.3%)	8 (5.1%)
Clinical and school assessments	0 (0%)	0 (0%)	0 (0%)	1 (7.1%)	1 (0.6%)
Medical and school records	1 (0.8%)	0 (0%)	0 (0%)	0 (0%)	1 (0.6%)
Medical records only	11 (9.3%)	3 (17.6%)	2 (25.0%)	2 (14.3%)	18 (11.5%)
Medical records and clinical assessments	3 (2.5%)	0 (0.0%)	1 (12.5%)	1 (7.1%)	5 (3.2%)
None described	71 (60.2%)	11 (64.7%)	4 (50.0%)	6 (42.9%)	92 (58.6%)
Parent information only	11 (9.3%)	0 (0%)	0 (0%)	1 (7.1%)	12 (7.6%)
Parent information and clinical assessments	10 (8.5%)	0 (0%)	1 (12.5%)	0 (0%)	11 (7.0%)
Parent information and medical records	5 (4.2%)	2 (11.8%)	0 (0%)	0 (0%)	7 (4.5%)
Parent information, clinical and school records	0 (0%)	0 (0%)	0 (0%)	1 (7.1%)	1 (0.6%)
Parent information and school records	1 (0.8%)	0 (0%)	0 (0%)	0 (0%)	1 (0.6%)
Total	118 (100.0%)	17 (100.0%)	8 (100.0%)	14 (100.0%)	157 (100.0%)

*Note:* Use of videos has not been included in this table.

### 3.4 Conditions Featuring Developmental Regression

The majority of studies described a neurodevelopmental disorder (*n* = 118, 75.2%). In this condition group, the conditions described were autism (*n* = 109, 92.4%); autism with Down syndrome (*n* = 1, 0.8%); childhood disintegrative disorder (*n* = 3, 2.5%); developmental regression without a known cause (*n* = 2, 1.7%); neurodevelopmental delay (*n* = 2, 1.7%); and autism with epilepsy (*n* = 1, 0.8%). Seventeen (10.8%) studies described progressive neurodegenerative conditions. Here, the conditions of interest were PIND (*n* = 4, 23.5%), metachromatic leukodystrophy (*n* = 3, 11.8%), CD (*n* = 2, 11.8%); and other neurodegenerative conditions (*n* = 8, 47.1%). For the eight (5.1%) studies of DEE conditions, five (62.5%) focused on epilepsy; one (12.5%) on autism; and two (25.0%) on continuous spike‐and‐wave during sleep. Of the 14 studies of genetic conditions, five (35.7%) focused on Rett syndrome; two on Phelan‐McDermid syndrome (14.3%); three (21.4%) on Down syndrome; two (14.3%) on *MECP2* duplication syndrome; one (7.1%) on Fragile X syndrome; and one (7.1%) on metachromatic leukodystrophy.

### 3.5 Studies by Year Group of Publication

**FIGURE 2:**
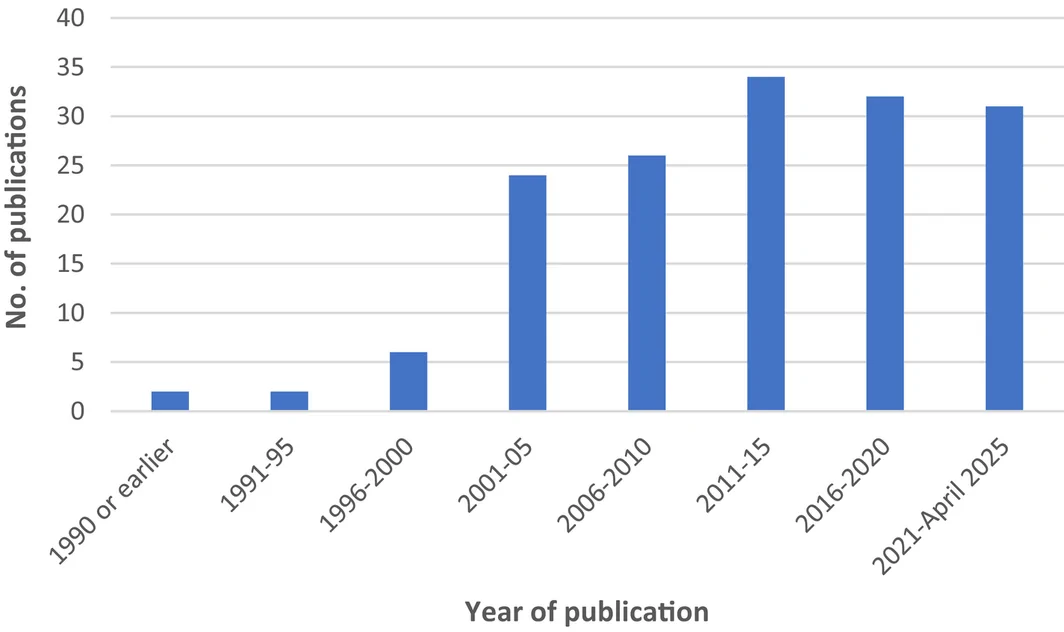
Number of papers by years of publication.

There has been a marked increase in studies focusing on developmental regression over the study period and particularly after 2000. Ten (6.4%) of the included studies were published in the years from 1983 to 2000, and from 2001 to 2010 there were 50 (31.8%) studies published. Finally, in the years from 2011 to April 2025, 97 (61.8%) of the included studies were published (Figure 2).

## 4 Discussion

Previous research (Furley et al. 2025; Zhang et al. 2019) has called for an agreed definition of developmental regression. This review adds to these calls by highlighting the inconsistency in the use of definitions and measures both within and between condition groups that feature developmental regression during childhood.

A major finding was that most studies of children experiencing developmental regression used an operational definition. However, these definitions varied in the reporting of fundamental features such as the duration of loss, the child's age when regression was first reported and the types of skills lost. Three papers (Aslan et al. 2021; Sandler et al. 2000; Yin et al. 2020) each provide examples. The first (Aslan et al. 2021) specified an age of onset before 3 years and a loss of more than five words for a period of more than 3 months. On the other hand, the second (Sandler et al. 2000) required the onset to be rapid and after 1 year of age and the duration to be less than four years. In contrast, the third (Yin et al. 2020) did not include onset and duration as features but defined developmental regression more generally as a *loss of acquired functions*.

The measurement of developmental regression using standardized and non‐standardized measures was also diverse in these three papers. For example, the first (Aslan et al. 2021) used parent information and medical records, while the second (Sandler et al. 2000) did not mention methods to measure developmental regression. For the third (Yin et al. 2020), descriptions in the proband's medical records established the occurrence of regression. Others (Goldberg et al. 2008; Maestro et al. 2006; Tonekaboni et al. 2025) used standardized measurements such as the ADI‐R, along with non‐standardized measures such as videos. In response to concerns that some developmental skill loss may be missed using the ADI‐R definition, two instruments were designed to supplement the ADI‐R: the RSF (Goldberg et al. 2003) and the RVI (Thurm et al. 2014). Yet few studies in this review used the RSF or the RVI in conjunction with the ADI‐R. Overall, in the review, we found that only a minority of studies used regression specific tools.

Except for studies of DEE, all condition groups used assessment scores over time as a method of measuring developmental regression. For example, a study (Thompson et al. 2019) used standardized measures to assess language and cognitive abilities at two time points in a cohort study of autistic children with and without regression. Whether declines in performance scores over time reflect true regression requires further scrutiny.

Study specific definitions and measures differing in fundamental features may change study participant inclusion, limit collaboration potential, hamper clinical interpretation and the translation of results. Measures used to capture skill loss need to minimize the negative influences of recall bias and telescoping that are inherent limitations of retrospective methods. Enquiring about the child's developmental abilities before the onset of regression, the timing and onset of regression, the duration of loss, the types of skills lost, the trajectory of loss, and information about concurrent events may provide important information for defining developmental regression.

Our review provides additional evidence that emphasizes the need for an agreed definition for developmental regression. Such a definition is needed for application at the onset of developmental regression when underlying causal mechanisms may not easily be identified. This would allow for earlier identification and assessment of a child with developmental regression. Transdiagnostic identification is essential given the breadth of conditions we now know feature developmental regression. As our knowledge grows, the advancement of regression phenotypes and associated diagnostic outcomes would likely enable their alignment with specific conditions. This systematic review provides the necessary foundations to further advance this field.

We found that the number of publications featuring developmental regression has increased markedly since 2000. With growing awareness of developmental regression, genomic expansions and new molecular findings associated with developmental regression (Furley et al. 2024), it is vital that studies and registries consistently define and measure developmental regression.

This study has notable strengths. We searched for any condition featuring developmental regression or a synonym or interchangeable term and performed detailed data extractions for the fundamental features used in definitions and measures. We completed a comprehensive search of the literature which allowed for rigorous, thorough, and accurate exploratory searching. Two researchers with experience in neurodevelopment screened each article for inclusion at all stages of the review to minimize error. The primary components used in definitions were categorized and conditions grouped to determine trends and patterns in the conditions, definitions, and measures used. We followed best practice guidelines using PRISMA‐Sc in alignment with the Joanna Briggs Institute.

Acknowledging limitations, this scoping review excluded case studies, reviews, and systematic reviews and meta‐analyses as these study designs were unlikely to report study definitions or fundamental features or measures of developmental regression. Despite extensive search terms, it is possible that some conditions featuring developmental regression were not identified through the search. Reaching an agreed definition for developmental regression will support future comprehensive and fully inclusive searches.

In order to improve our understanding of developmental regression and its objective assessment, future studies are needed to investigate the abilities of the child prior to regression and the objective analysis of home videos in the assessment of regression. Examining the defining of developmental regression by year of publication would improve our understanding of how definitions and measures have changed over time.

We did not evaluate reported pre‐loss abilities. Categorizing conditions into groups such as neurodevelopmental, neurodegenerative, genetic, and DEE was intended to determine whether there were differences in how studies define and measure by the type of condition. We acknowledge that there are significant overlaps between these categories and therefore such groupings cannot be precise. Finally, this is a scoping review of the literature. Hence, it was not possible to review the details of every study‐specific questionnaire, or to report age of onset, skill type, or duration for every study. Further research to consider these fundamental features at the individual condition or individual study level is recommended.

Neurodevelopmental disorders comprised more than three quarters of conditions investigated in terms of the defining and measuring of developmental regression. Within this condition group, autism was the primary condition explored in almost all of the papers. The rising prevalence of autism with developmental regression, estimated as nearly 1% in 2022, emphasizes the increasing importance of working towards a standardized definition of developmental regression and establishing optimal methods for its measurement.

## 5 Conclusions

Our results highlight important fundamental features to consider when defining a child with symptoms of developmental regression. These include the age of the child at the onset of the regression, the types of skills lost, the duration of the loss, and whether any concurrent events occurred at the onset of regression. Subsequently, these results have informed the establishment of eligibility criteria for a novel clinical service for children experiencing developmental regression and have provided a foundation for further research to reach an agreed definition. For example, a consensus‐seeking study is currently underway which draws on the fundamental features identified in this review with the aim of proposing an agreed approach to define and measure developmental regression.

## Author Contributions

**Kirsten Furley:** conceptualized the study, developed the search strategy, completed screening and data extraction, and data analysis, wrote and edited the manuscript. **Katrina Williams:** conceptualized the study, supervised Dr. Furley in developing the search strategy, helped with data extraction, and editing. **Amanda Brignell:** supported the conceptualisation of the study, helped with screening and data extraction, and editing the manuscript. **Jenny Fairthorne:** screened, extracted, and analyzed data and assisted with the writing and editing of the final manuscript. **Vishnu Priya Mohanakumar Sindhu:** screened and extracted data and helped with editing the manuscript.

## Funding

This work was supported by Monash University.

## Conflicts of Interest

The authors declare no conflicts of interest.

## Supporting information


**Table S1:** Included papers and their properties.

## Data Availability

The data that support the findings of this study are available from the corresponding author upon reasonable request.
