# Supplementary material for: Defining and Measuring Developmental Regression During Childhood: A Scoping Review
Source: Autism Res. 2026 Aug 8;19(9):e70325. doi: 10.1002/aur.70325 (PMC13599686; doi:10.1002/aur.70325)
Supplement: Supplementary file 1 [file AUR-19-0-s001.docx]

Supplementary Table 1: Included papers and their properties

|  | | | **Definition & features of regression provided by paper** | | | | | **Measures of the regression** | |
| --- | --- | --- | --- | --- | --- | --- | --- | --- | --- |
| **Citation** | **Condition group** | **Primary condition** | **Operational definition** | **Duration** | **Onset** | **Types of skills lost** | **Key measure(s)** | | **Study specific**  **measure** |
| Paper 1. (1) | Progressive neurodegenerative conditions | Juvenile Neuronal Ceroid Lipofuscinosis | No | No | Yes | Mixed | Unified Batten Disease Rating Scale | | No |
| Paper 2. (2) | Progressive neurodegenerative conditions | EO metachromatic leukodystrophy | Yes | No | Yes | Mixed | Medical records | | No |
| Paper 3. (3) | Neurodevelopmental disorders | Autism | Yes | Yes | Yes | Language & social | Video, AuBC, ABC, CARS, ADST | | No |
| Paper 4. (4) | Neurodevelopmental disorders | Autism | Yes | Yes | Yes | Language | ADI-R, ADOS, MSEL, VABS-II | | No |
| Paper 5. (5) | Neurodevelopmental disorders | Autism | Yes | Yes | Yes | Language | ADI-R, ADOS, MSEL, VABS-II | | No |
| Paper 6. (6) | Neurodevelopmental disorders | Autism | Yes | No | Yes | Language & social | ADI-R, ADOS-G | | No |
| Paper 7. (7) | Neurodevelopmental disorders | Autism | Yes | No | Yes | Mixed | ADI-R, SCQ, DISCO, CPM, VABS, medical records | | No |
| Paper 8. (8) | Neurodevelopmental disorders | Autism | Yes | Yes | Yes | Mixed | Parent interviews, CARS | | No |
| Paper 9. (9) | Genetic conditions | Down syndrome | Yes | Yes | Yes | Mixed | ABC | | No |
| Paper 10. (10) | Neurodevelopmental disorders | Autism | No | No | Yes | Mixed | ADI-R, ADOS-2, CARS, LT, CGAS | | No |
| Paper 11. (11) | Neurodevelopmental disorders | Autism | Yes | Yes | Yes | Mixed | SCQ, RSQ, EDQ, WNV, ADI-R, ADOS | | No |
| Paper 12. (12) | Neurodevelopmental disorders | Autism | Yes | No | Yes | Mixed | Medical records | | No |
| Paper 13. (13) | Neurodevelopmental disorders | Autism | Yes | No | Yes | Language & social | VABS-II, Preschool Lang. Scale, PPVT. | | No |
| Paper 14. (14) | Neurodevelopmental disorders | Autism | No | No | No | Not described | Study specific measure | | Parent questionnaire |
| Paper 15. (15) | Genetic conditions | Rett syndrome | No | Yes | Yes | Mixed | Medical records | | No |
| Paper 16. (16) | Genetic conditions | Rett syndrome | No | No | Yes | Mixed | Video | | No |
| Paper 17. (17) | Neurodevelopmental disorders | Autism | Yes | Yes | No | Mixed | Neurologic & parent reports, WISC-R, SPM, LT | | No |
| Paper 18. (18) | Neurodevelopmental disorders | Autism & Down syndrome | Yes | Yes | Yes | Mixed | ADI-R, ADOS | | No |
| Paper 19. (19) | Neurodevelopmental disorders | Autism | Yes | No | Yes | Language | Parent reports, WISC-R, SPM, LT, clinical & phone interviews | | No |
| Paper 20. (20) | Neurodevelopmental disorders | Autism |  | No | Yes | Language & social | ADI-R | | No |
| Paper 21. (21) | Neurodevelopmental disorders | Autism | No | No | Yes | Language & social | Parent questionnaire | | No |
| Paper 22. (22) | Neurodevelopmental disorders | Autism | Yes | Yes | Yes | Mixed | AuBC | | No |
| Paper 23. (23) | Genetic conditions | Phelan-McDermid syndrome | Yes | No | Yes | Language & motor | Inpatient stays, medical records, questionnaire | | No |
| Paper 24. (24) | Progressive neurodegenerative conditions | Childhood dementia | No | Yes | Yes | Mixed | Parents’ Visual Analogue of Pain Scale | | No |
| Paper 25. (25)*…* | Developmental epileptic encephalopathies | Epilepsy | No | No | No | Cognitive | WISC-III, WPPSI-R, medical records | | No |
| Paper 26. (26) | Neurodevelopmental disorders | Autism | Yes | No | No | Mixed | Clinical assessment | | No |
| Paper 27. (27) | Neurodevelopmental disorders | Autism | Yes | Yes | Yes | Mixed | Study specific measure, video | | Semi-structured enquiry |
| Paper 28. (28) | Neurodevelopmental disorders | Childhood disintegrative disorder | No | No | No | Language & social | Study specific measure | | Questionnaire/ Interview guide |
| Paper 29. (29) | Neurodevelopmental disorders | Autism | Yes | No | Yes | Mixed | ASD-Child, ASD-DC | | No |
| Paper 30. (30) | Developmental epileptic encephalopathies | Epilepsy | No | No | Yes | Not described | CAQ, BDQ | | No |
| Paper 31. (31) | Neurodevelopmental disorders | Autism | Yes | Yes | Yes | Mixed | ADI-R, BSID-C, PPVT, parent reports | | No |
| Paper 32. (32) | Neurodevelopmental disorders | Autism | Yes | No | Yes | Mixed | ADI, WPPSI, The Merrill-Palmer | | No |
| Paper 33. (33) | Neurodevelopmental disorders | Autism | Yes | Yes | Yes | Mixed | ADOS, ADI-R, DANR | | No |
| Paper 34. (34) | Neurodevelopmental disorders | Autism | No | No | No | Not described | ABC, SRS, DANR, ADOS | | No |
| Paper 35. (35) | Neurodevelopmental disorders | Developmental regression | No | No | No | Mixed | Not described | | No |
| Paper 36. (36) | Neurodevelopmental disorders | Autism | Yes | No | Yes | Mixed | Clinician interview, CASI-4R, parent questionnaire | | No |
| Paper 37. (37) | Neurodevelopmental disorders | Autism | Yes | Yes | Yes | Language | ADI-R, ADOS, VABS, PPVT, DAS, MSEL, WISC | | No |
| Paper 38. (38) | Neurodevelopmental disorders | Autism | Yes | No | No | Language & social | Medical records | | No |
| Paper 39. (39) | Neurodevelopmental disorders | Autism | Yes | No | No | Language & social | ADOS-2 | | No |
| Paper 40. (40) | Neurodevelopmental disorders | Autism | Yes | No | Yes | Mixed | Parent checklist; CBC | | No |
| Paper 41. (41) | Neurodevelopmental disorders | Autism | Yes | Yes | Yes | Mixed | ADI-R, ADOS-G, CARS, GMDS, Leiter IPS, CSHQ | | No |
| Paper 42. (42) | Neurodevelopmental disorders | Autism | Yes | Yes | Yes | Mixed | ADI-R, ADOS, Leiter IPS, CBC, CSHQ, WPPSI, WISC | | No |
| Paper 43. (43) | Neurodevelopmental disorders | Autism | Yes | No | No | Language & social | Clinical assessment | | No |
| Paper 44. (44) | Neurodevelopmental disorders | Autism | No | No | Yes | Mixed | Study specific measure | | Parent questionnaire |
| Paper 45. (45) | Neurodevelopmental disorders | Autism | Yes | Yes | Yes | Mixed | ADI-R, ADOS, MSEL, DAS, WISC, parent interview | | No |
| Paper 46. (46) | Neurodevelopmental disorders | Autism | Yes | No | Yes | Mixed | ADI-R, EDQ, IPQ-R adapted for autism. | | No |
| Paper 47. (47) | Neurodevelopmental disorders | Autism | Yes | No | No | Mixed | ADI-R, ADOS, parent interview | | No |
| Paper 48. (48) | Neurodevelopmental disorders | Autism | Yes | Yes | Yes | Mixed | ADI-R, video, RSF, ADOS, SBT, MSEL | | No |
| Paper 49. (49) | Neurodevelopmental disorders | Autism | Yes | No | No | Mixed | ADI-R, video, RSF | | No |
| Paper 50. (50) | Genetic conditions | Rett syndrome | Yes | Yes | Yes | Mixed | Clinician assessment | | No |
| Paper 51. (51) | Genetic conditions | Fragile X syndrome | Yes | No | Yes | Mixed | VABS | | No |
| Paper 52. (52) | Neurodevelopmental disorders | Autism | Yes | Yes | Yes | Mixed | ADOS, ACHQ, study specific measure | | Parent questionnaire |
| Paper 53. (53) | Neurodevelopmental disorders | Autism | Yes | No | No | Language & social | ADI-R, ADOS | | No |
| Paper 54. (54) | Neurodevelopmental disorders | Developmental regression | No | No | No | Language & social | Study specific measure | | Checklist from M-CHAT & SCQ. |
| Paper 55. (55) | Neurodevelopmental disorders | Autism | Yes | No | Yes | Mixed | Study specific measure, interviews by clinicians | | Interview questionnaire |
| Paper 56. (56) | Neurodevelopmental disorders | Autism | Yes | No | No | Mixed | Questionnaire based on SCQ, ADI-R, CARS, parent interview | | No |
| Paper 57. (57) | Neurodevelopmental disorders | Autism | Yes | Yes | Yes | Mixed | ACHQ, AuBC, SRS, CARS | | No |
| Paper 58. (58) | Neurodevelopmental disorders | Autism | Yes | Yes | Yes | Mixed | Study specific measure, parent interview, SRS, CARS | | Parent questionnaire |
| Paper 59. (59) | Neurodevelopmental disorders | Neurodevelopmental delay | No | No | No | Mixed | Not described | | No |
| Paper 60.(60) | Neurodevelopmental disorders | Autism | Yes | No | Yes | Mixed | DST, M-CHAT, study specific measure, , | | Parent-Rating Checklist |
| Paper 61. (61) | Neurodevelopmental disorders | Autism | Yes | No | Yes | Language | ADI-R, ADOS, parent interview & clinical assessment | | No |
| Paper 62. (62) | Neurodevelopmental disorders | Autism | No | No | No | Not described | Parent reports | | No |
| Paper 63. (63) | Neurodevelopmental disorders | Autism | Yes | Yes | Yes | Language | CARS, AuBC, study specific measure | | Form for parents. |
| Paper 64. (64) | Neurodevelopmental disorders | Autism | Yes | No | Yes | Not described | IAN Project questionnaire | | No |
| Paper 65. (65) | Neurodevelopmental disorders | Neurodevelopmental delay | No | No | No | Mixed | Not described | | No |
| Paper 66. (66) | Progressive neurodegenerative conditions | PIND | Yes | Yes | No | Mixed | Study specific measure | | Physician questionnaire |
| Paper 67. (67) | Progressive neurodegenerative conditions | Metachromatic leukodystrophy | No | Yes | Yes | Motor | Study specific measure, video | | Patient questionnaire |
| Paper 68. (68) | Progressive neurodegenerative conditions | Metachromatic leukodystrophy | Yes | Yes | Yes | Mixed | Study specific measure | | Patient questionnaire |
| Paper 69. (69) | Neurodevelopmental disorders | Autism | Yes | No | Yes | Mixed | CARS, M-CHAT | | No |
| Paper 70. (70) | Neurodevelopmental disorders | Autism | Yes | No | Yes | Mixed | Clinical assessment | | No |
| Paper 71. (71) | Genetic conditions | Phelan-McDermid syndrome | Yes | Yes | No | Mixed | Study specific measure | | Semi-structured interview. |
| Paper 72. (72) | Neurodevelopmental disorders | Autism | Yes | No | Yes | Mixed | Study specific measure | | Structured questionnaire |
| Paper 73. (73) | Neurodevelopmental disorders | Childhood disintegrative disorder | Yes | No | Yes | Mixed | Questionnaire, clinical assessment & records | | No |
| Paper 74. (74) | Neurodevelopmental disorders | Autism | Yes | Yes | No | Language | SBT, Parent questionnaire | | No |
| Paper 75. (75) | Neurodevelopmental disorders | Childhood disintegrative disorder | Yes | No | No | Mixed | Clinical records & assessment, questionnaire | | No |
| Paper 76. (76) | Neurodevelopmental disorders | Autism | Yes | Yes | Yes | Language | ADI-R | | No |
| Paper 77. (77) | Neurodevelopmental disorders | Autism | No | Yes | Yes | Mixed | Data collection forms, parent interview & CGAS | | No |
| Paper 78. (78) | Genetic conditions | Rett syndrome | Yes | No | Yes | Mixed | ARSD, InterRett, phone interview, video, photos | | No |
| Paper 79. (79) | Neurodevelopmental disorders | Autism | Yes | Yes | Yes | Mixed | CARS, parent interview | | No |
| Paper 80. (80) | Neurodevelopmental disorders | Autism | Yes | No | Yes | Language | ADI-R, video | | No |
| Paper 81. (81) | Neurodevelopmental disorders | Autism | Yes | Y | Y | Language & social | ADI-R, Weschler assessments, ADOS | | No |
| Paper 82. (82) | Neurodevelopmental disorders | Autism | Yes | No | No | Mixed | ADI-R, video, Grid for attention in Infants | | No |
| Paper 83. (83) | Neurodevelopmental disorders | Autism | Yes | No | Yes | Mixed | ADI-R | | No |
| Paper 84. (84) | Neurodevelopmental disorders | Autism | Yes | No | Yes | Mixed | DPII, VSMS, CARS | | No |
| Paper 85. (85) | Neurodevelopmental disorders | Autism | Yes | No | Yes | Mixed | Study specific measure, ADOS, medical records | | Intake questionnaire |
| Paper 86. (86) | Genetic conditions | *MECP2* duplication syndrome | No | No | No | Not described | Study specific measure, medical records | | Parent questionnaire |
| Paper 87. (87) | Neurodevelopmental disorders | Autism | Yes | No | No | Language & social | Questionnaire from ADI-R, BDT, ADOS & PDDBI parent rating | | No |
| Paper 88. (88) | Neurodevelopmental disorders | Autism | Yes | No | Yes | Mixed | Study specific measure | | Demographic questionnaire |
| Paper 89. (89) | Developmental epileptic encephalopathies | Epilepsy | Yes | No | No | Language | Medical & other records | | No |
| Paper 90. (90) | Neurodevelopmental disorders | Autism | Yes | Yes | No | Mixed | ADI-R | | No |
| Paper 91. (91) | Neurodevelopmental disorders | Autism | Yes | No | No | Mixed | ADI-R, SRS, teacher ratings, MSEL, DAS-II, WISC-IV, parent information | | No |
| Paper 92. (92) | Neurodevelopmental disorders | Autism | Yes | Yes | No | Language | ADI-R, ADOS, DAS, MSEL | | No |
| Paper 93. (93) | Progressive neurodegenerative conditions | Late-infantile neuronal ceroid lipofuscinosis type 2 disease | No | No | No | Mixed | Hamburg CLN2 scale, Weill Cornell CLN2 scale | | No |
| Paper 94. (94) | Neurodevelopmental disorders | Autism | Yes | No | No | Language | ADI-R, ADOS, MSEL | | No |
| Paper 95. (95) | Progressive neurodegenerative conditions | Childhood dementia | Yes | Yes | No | Mixed | Study specific measure, medical records | | CD questionnaire |
| Paper 96. (96) | Neurodevelopmental disorders | Autism | No | Yes | Yes | Mixed | CARS, GDS, CAST, IQ tests | | No |
| Paper 97. (97) | Neurodevelopmental disorders | Autism | Yes | Yes | Yes | Language & social | ADI-R, EDQ, ADOS | | No |
| Paper 98. (98) | Neurodevelopmental disorders | Autism | Yes | No | No | Language & social | ADI-R, video, ADOS, MSEL, VABS | | No |
| Paper 99. (99) | Neurodevelopmental disorders | Autism | Yes | Yes | Yes | Language & social | ADI-R, video | | No |
| Paper 100. (100) | Neurodevelopmental disorders | Autism | Yes | No | No | Language & social | ADI-R, EDQ, study specific measure | | Session summary tool |
| Paper 101. (101) | Neurodevelopmental disorders | Autism | Yes | No | Yes | Language | ADI-R, ADOS, VABS, RVI | | No |
| Paper 102. (102) | Neurodevelopmental disorders | Autism | Yes | Yes | Yes | Mixed | ADI-R, VABS, SPM, MSEL, BPVT, PPVT, medical records | | No |
| Paper 103. (103) | Progressive neurodegenerative conditions | Juvenile neuronal ceroid lipofuscinosis | Yes | No | Yes | Mixed | Study specific measure, medical records, parent interviews | | Database with 50 items |
| Paper 104. (104) | Progressive neurodegenerative conditions | Late infantile neuronal ceroid lipofuscinosis | No | No | Yes | Mixed | Study specific measure, medical records, parent reports | | As above |
| Paper 105. (105) | Genetic conditions | *MECP2* duplication syndrome | Yes | No | Yes | Mixed | Clinical assessment, ADI-R, VABS-II, BSID-III, ADOS | | No |
| Paper 106. (106) | Neurodevelopmental disorders | Autism | Yes | Yes | Yes | Language | ADI-R, ADOS-G, SPM, IQ measures | | No |
| Paper 107. (107) | Neurodevelopmental disorders | Autism | Yes | Yes | Yes | Not described | ADI-R, PDDBI, ADOS-R, BDT | | No |
| Paper 108. (108) | Developmental epileptic encephalopathies | Epilepsy | No | No | No | Not described | Medical records, hospital database | | No |
| Paper 109. (109) | Neurodevelopmental disorders | Autism | Yes | No | Yes | Mixed | ADI-R, ADOS, VABS, ADOS-T | | No |
| Paper 110. (110) | Neurodevelopmental disorders | Autism | No | No | Yes | Language & social | AIIMS-Mod-INDT, ADI-R, MSEL, CARS | | No |
| Paper 111. (111) | Neurodevelopmental disorders | Autism | Yes | No | Yes | Language & social | ADOS-2, VABS-II, GMDS-ER, CBC, RBS-R, SCQ | | No |
| Paper 112. (112) | Neurodevelopmental disorders | Autism | Yes | Yes | Yes | Cognitive | ADI-R, ADOS-R, PDDBI, BDT | | No |
| Paper 113. (113) | Neurodevelopmental disorders | Autism | Yes | No | Yes | Language & social | ADI-R, VABS, CBC, ADOS, Carer Interview, MSEL | | No |
| Paper 114. (114) | Neurodevelopmental disorders | Autism | Yes | Yes | Yes | Mixed | ADI-R, ADOS … | | No |
| Paper 115. (115) | Progressive neurodegenerative conditions | Infantile & childhood onset PLA2G6 | No | No | No | Mixed | Not described | | No |
| Paper 116. (116) | Neurodevelopmental disorders | Autism | Yes | Yes | Yes | Mixed | DPII, CARS, video | | No |
| Paper 117. (117) | Genetic conditions | Down syndrome | Yes | No | Yes | Mixed | Family & school information, video, medical records | | No |
| Paper 118. (118) | Developmental epileptic encephalopathies | CSWS | Yes | No | Yes | Language | Receptive-expressive Emergent Lang. Scale … | | No |
| Paper 119. (119) | Neurodevelopmental disorders | Autism | Yes | No | Yes | Mixed | ADI-R, medical records | | No |
| Paper 120. (120) | Developmental epileptic encephalopathies | CSWS | Yes | No | Yes | Cognitive | Various neuro-psychological tests | | No |
| Paper 121. (121) | Genetic conditions | Mucopolysaccharidosis Type IIIA | Yes | No | Yes | Mixed | VABS-II, BSID, 4PSS, CSHQ | | No |
| Paper 122. (122) | Progressive neurodegenerative conditions | Lysosomal Storage Disorder | Yes | No | No | Mixed | Not described | | No |
| Paper 123. (123) | Neurodevelopmental disorders | Epilepsy | Yes | No | Yes | Language | Parent interview, clinical assessment | | No |
| Paper 124. (124) | Neurodevelopmental disorders | Autism | No | No | No | Language & social | Chart review | | No |
| Paper 125. (125) | Neurodevelopmental disorders | Autism | Yes | No | Yes | Not described | ADI-R, ADOS, MSEL, VABS-II | | No |
| Paper 126. (126) | Neurodevelopmental disorders | Autism | No | No | No | Not described | OACIS, ABC, SRS, parent question naire, medical records & tests | | No |
| Paper 127. (127) | Neurodevelopmental disorders | Autism | No | No | Yes | Mixed | Study specific measure, medical records & assessments | | Parent questionnaire |
| Paper 128. (128) | Progressive neurodegenerative conditions | Leukodystrophies | Yes | Yes | Yes | Mixed | Checklist for recruiting pediatricians | | No |
| Paper 129. (129) | Neurodevelopmental disorders | Autism | Yes | No | Yes | Language & social | SBT, clinical assessment, parent reports | | No |
| Paper 130. (130) | Neurodevelopmental disorders | Autism | Yes | No | Yes | Language& social | WPPSI-III, Griffiths' test, parent interview, medical records | | No |
| Paper 131. (131) | Neurodevelopmental disorders | Autism | Yes | No | Yes | Mixed | Health registers, medical records | | No |
| Paper 132. (132) | Neurodevelopmental disorders | Autism | Yes | No | Yes | Mixed | Clinical assessment, ABC, ADOS-G, medical records, | | No |
| Paper 133. (133) | Neurodevelopmental disorders | Autism | Yes | No | Yes | Mixed | ADI-R, ADOS, MSEL, DAS-II | | No |
| Paper 134. (134) | Progressive neurodegenerative conditions | Leigh syndrome spectrum disorder | Yes | No | Yes | Mixed | Medical records | | No |
| Paper 135. (135) | Neurodevelopmental disorders | Autism | Yes | No | Yes | Mixed | ADI-R, CBC, SCQ, Adaptive Behavior Assessment System | | No |
| Paper 136. (136) | Neurodevelopmental disorders | Autism | No | No | Yes | Not described | DISCO | | No |
| Paper 137. (137) | Developmental epileptic encephalopathies | Autism | Yes | No | Yes | Language | Clinical assessment, parent reports | | No |
| Paper 138. (138) | Neurodevelopmental disorders | Autism | Yes | Yes | No | Mixed | Study specific measure, clinical assessment, interviews… | | Intake questionnaire |
| Paper 139. (139) | Genetic conditions | Rett syndrome | No | No | No | Language | Questionnaires from ARSD & InterRett Database | | No |
| Paper 140. (140) | Neurodevelopmental disorders | Autism | Yes | No | No | Language | Parent interview, CARS, medical records | | No |
| Paper 141. (141) | Developmental epileptic encephalopathies | Epilepsy | Yes | Yes | No | Cognitive | Neurological/neuro-psychological reports | | No |
| Paper 142. (142) | Progressive neurodegenerative conditions | PIND | Yes | Yes | Yes | Mixed | PIND tool | | No |
| Paper 143. (143) | Progressive neurodegenerative conditions | PIND | Yes | No | No | Mixed | PIND tool | | No |
| Paper 144. (144) | Progressive neurodegenerative conditions | PIND | Yes | Yes | Yes | Mixed | PIND tool | | No |
| Paper 145. (145) | Neurodevelopmental disorders | Autism | Yes | No | No | Language & social | Clinical assessment, personal health books | | No |
| Paper 146. (146) | Neurodevelopmental disorders | Autism | Yes | No | Yes | Mixed | ADI-R, ADOS-G | | No |
| Paper 147.(147) | Neurodevelopmental disorders | Autism | Yes | No | Yes | Mixed | ADI-R, ADOS-G, video, EDI, MSEL | | No |
| Paper 148. (148) | Neurodevelopmental disorders | Autism | Yes | No | No | Mixed | Education & health data, IQ score | | No |
| Paper 149. (149) | Neurodevelopmental disorders | Autism | Yes | Yes | Yes | Language | Medical records | | No |
| Paper 150. (150) | Neurodevelopmental disorders | Autism | Yes | No | No | Mixed | ADI-R, study specific measure, medical records & testing | | Survey questionnaire |
| Paper 151. (151) | Genetic conditions | Down syndrome | Yes | No | No | Mixed | Clinical assessment, school records | | No |
| Paper 152. (152) | Neurodevelopmental disorders | Autism | Yes | No | Yes | Language & social | ADOS, clinical assessment | | No |
| Paper 153. (153) | Neurodevelopmental disorders | Autism | Yes | No | No | Mixed | Medical records | | No |
| Paper 154. (154) | Neurodevelopmental disorders | Autism | Yes | Yes | No | Language & social | ADI-R, CARS, ABC, Sociodemographic Data Form, RBS | | No |
| Paper 155. (155) | Neurodevelopmental disorders | Autism | No | No | No | Language & social | ABC, Portage Checklist | | No |
| Paper 156. (156) | Neurodevelopmental disorders | Autism | Yes | Yes | Yes | Language & social | Parent interview & medical records | | No |
| Paper 157. (157) | Neurodevelopmental disorders | Autism | Yes | Yes | No | Language | ABC, Psycho-educational Profile, parent reports, | | No |

Abbreviations: 4PSS, Four Point Scoring System; ABC, Aberrant Behavior Checklist; ADI-R, Autism Diagnostic Interview –Revised; ACHQ; ASD Child Health Questionnaire; ADOS, Autism Diagnostic Observation Schedule; ADST, Ankara Developmental Screening Test; AIIMS-Mod-INDT, All India Institute of Medical Sciences-Modified-INCLEN diagnostic tool; ARSD, Australian Rett Syndrome Database; ASD-Child, Autism Spectrum Disorders Assessment Battery for Children; ASD-DC, AuBC, Autism Behaviour Checklist; Autism, Autism spectrum disorder; AuBC, Autism Behavior Checklist; BDT, Battelle Development Test; BDQ, Birth and ASD Diagnosis History Questionnaire; BPVT, British Picture Vocabulary Test; BSID; Bayley Scales of Infant and Toddler Development; CAQ, Child with ASD Questionnaire; CARS, Childhood Autism Rating Scale; CASI-4R, Child and Adolescent Symptom Inventory-4R; CAS, Childhood Asperger’s Syndrome Test; CBC, Child Behavior Checklist; CD, Childhood dementia; CELF, Clinical Evaluation of Language Fundamentals; CGAS, Children's Global Assessment Scale; CLN2, Neuronal ceroid lipofuscinosis; CPM, Coloured Progressive Matrices; CSHQ, Children’s Sleep Habits Questionnaire; CSWS, Continuous spike-and-wave during sleep; DANR, Developmental and Neurobehavioral Regression Questionnaire; DAS; Differential Ability Scales; DISCO, Diagnostic Interview for Social and Communication Disorders; DPII, DST, Developmental Screening Test; EDQ, Early Development Questionnaire; EO, Early onset; GDS, Gesell Developmental Scale; GI, Gastrointestinal; GMDS-ER, Griffiths Mental Development Scales-Extended Revised; GRSF, Goldberg Regression Supplementation Form; IAN, Interactive Autism Network; INCLEN, International Clinical Epidemiology Network; InterRett, International Rett Syndrome Phenotype Database; IPQ-R, Revised Illness Perception Questionnaire; IPS, International Performance Scale; IQ, Intelligence quotient; Lang., Language; LT, Leiter test; M-ABC, Movement Assessment Battery for Children; M-CHAT, Modified Checklist for Autism in Toddlers; MSEL, Mullen Scales of Early Learning; OACIS-S, Ohio Autism Clinical Impression Scale–Severity; O/C, outcome; PDDBI, Pervasive Developmental Disorder Behavior Inventory; PIND, Progressive intellectual and neurological deterioration; PPVT, Peabody Picture Vocabulary Test; RBS, Repetitive Behavior Scale; RSF, Regression Supplement Form; RSQ, Regression Supplemental Questions; SBT, Stanford-Binet IQ Test, SCQ, Social Communication Questionnaire; SPM, Raven’s Standard Progressive Matrices; SRS, Social Responsiveness Scale; syn syndrome; VABS, Vineland Adaptive Behavior Scale; VSMS, Vineland Social Maturity Scale; Vs, Versus; WISC, Wechsler Intelligence Scale for Children; WNV, Wechsler Non-Verbal Scale of Ability; WPPSI, Wechsler Preschool and Primary Scale of Intelligence.

Note: When a version of a tool is referenced in the table, only the acronym of the tool and not the version is listed as an abbreviation.

**References**

1.	Adams HR, Mink JW, University of Rochester Batten Center Study G. Neurobehavioral features and natural history of juvenile neuronal ceroid lipofuscinosis (Batten disease). Journal of child neurology. 2013;28(9):1128-36.

2.	Adang LA, Groeschel S, Grzyb C, D'Aiello R, Gavazzi F, Sherbini O, et al. Developmental delay can precede neurologic regression in early onset metachromatic leukodystrophy. Molecular Genetics & Metabolism. 2024;142(4):108521.

3.	Aslan C, Konuşkan B, Şener B, Ünal F. Comparison of serum anti-neuronal antibody levels in patients having autism spectrum disorder with and without regression. The Turkish Journal of Pediatrics. 2021;63(5):780-9.

4.	Backes B, Zanon RB, Bosa CA. The relation between language regression and social communicative development of children with autism spectrum disorder. CoDAS. 2013;25(3):268-73.

5.	Backes B, Zanon RB, Bosa CA. Symptomatic Characteristics of Children with Autism and Language Regression. Psicologia: Teoria e Pesquisa. 2017;33:e3343.

6.	Baird G, Robinson RO, Boyd S, Charman T. Sleep electroencephalograms in young children with autism with and without regression. Developmental Medicine and Child Neurology. 2006;48(7):604-8.

7.	Baird G, Charman T, Pickles A, Chandler S, Loucas T, Meldrum D, et al. Regression, developmental trajectory and associated problems in disorders in the autism spectrum: the SNAP study. J Autism Dev Disord. 2008;38(10):1827-36.

8.	Bernabei P, Cerquiglini A, Cortesi F, D’ardia C. Regression versus no regression in the autistic disorder: Developmental trajectories. J Autism Dev Disord. 2007;37:580-8.

9.	Bernad-Ripoll S, O'Neill M, Capone G. Children with Down syndrome who experience developmental skill loss, characterization, and phenomenology: A case series. Am J Med Genet, Part C: . 2023;193(4).

10.	Bolognesi E, Guerini FR, Carta A, Chiappedi M, Sotgiu S, Mensi MM, et al. The role of SNAP-25 in autism spectrum disorders onset patterns. Int J Mol Sci. 2023;24(18):14042.

11.	Boterberg S, Charman T, Marschik PB, Bölte S, Roeyers H. Regression in autism spectrum disorder: A critical overview of retrospective findings and recommendations for future research. Neuroscience & Biobehavioral Reviews. 2019;102:24-55.

12.	Bradley CC, Boan AD, Cohen AP, Charles JM, Carpenter LA. Reported history of developmental regression and restricted, repetitive behaviors in children with autism spectrum disorders. Journal of Developmental & Behavioral Pediatrics. 2016;37(6):451-6.

13.	Brignell A, Williams K, Prior M, Donath S, Reilly S, Bavin EL, et al. Parent-reported patterns of loss and gain in communication in 1-to 2-year-old children are not unique to autism spectrum disorder. Autism. 2017;21(3):344-56.

14.	Brown J, Prelock PA. Brief report: the impact of regression on language development in autism. J Autism Dev Disord. 1995;25(3).

15.	Bruck I, Antoniuk SA, Halick SM, Spessatto A, Bruyn LR, Rodrigues M, et al. Rett syndrome: retrospective and prospective study of 28 patients. Arq Neuropsiquiatr. 2001;59(2-b):407-10.

16.	Burford B, Kerr AM, Macleod HA. Nurse recognition of early deviation in development in home videos of infants with Rett disorder. J Intellect Disabil Res. 2003;47(Pt 8):588-96.

17.	Canitano R, Luchetti A, Zappella M. Epilepsy, electroencephalographic abnormalities, and regression in children with autism. J Child Neurol. 2005;20(1):27-31.

18.	Castillo H, Patterson B, Hickey F, Kinsman A, Howard JM, Mitchell T, et al. Difference in age at regression in children with autism with and without Down syndrome. J Dev Behav Pediatr. 2008;29(2):89-93.

19.	Christopher JA, Sears LL, Williams PG, Oliver J, Hersh J. Familial, medical and developmental patterns of children with autism and a history of language regression. Journal of Developmental and Physical Disabilities. 2004;16(2):163-70.

20.	D'Ardia C. Autism with regression vs. autism without regression: a clinical comparison. Psicologia clinica dello sviluppo. 2003;7(1):119 - 33.

21.	Davidovitch M, Glick L, Holtzman G, Tirosh E, Safir MP. Developmental regression in autism: maternal perception. J Autism Dev Disord. 2000;30(2):113-9.

22.	Devecioglu H, Tan C, Misir E, Esen H, Ozbek B, Kultur S. Investigation of Neuronal-Astroglial Injury Proteins and MMP-9 Serum Levels in autism spectrum disorder and their relationship with autistic regression. International Journal of Developmental Neuroscience 2025;85(1):113–9.

23.	Dille Y, Lagae L, Swillen A, Buggenhout GV. Neurodevelopmental profile and stages of regression in Phelan–McDermid syndrome. Developmental Medicine & Child Neurology. 2023;65(7):917-25.

24.	Djafar J, Johnson A, Elvidge K, Farrar M. Childhood dementia: A collective clinical approach to advance therapeutic development and care. Pediatric Neurology. 2023;139:76-85.

25.	Dorris L, O'Regan M, Wilson M, Zuberi SM. Progressive intellectual impairment in children with Encephalopathy related to Status Epilepticus during slow Sleep. Epileptic Disord. 2019;21(S1):88-96.

26.	Duffy FH, Shankardass A, McAnulty GB, Eksioglu YZ, Coulter D, Rotenberg A, et al. Corticosteroid therapy in regressive autism: a retrospective study of effects on the Frequency Modulated Auditory Evoked Response (FMAER), language, and behavior. BMC Neurol. 2014;14:70.

27.	Ekinci O, Arman AR, Melek I, Bez Y, Berkem M. The phenomenology of autistic regression: subtypes and associated factors. European Child & Adolescent Psychiatry. 2012;21(1):23-9.

28.	Ellis MJ, Larsen K, Havighurst SS. Childhood Disintegrative Disorder (CDD): Symptomatology of the Norwegian patient population and parents’ experiences of patient regression. J Autism Dev Disord. 2022;52(4):1495-506.

29.	Estabillo JA, Matson JL, Cervantes PE. Autism symptoms and problem behaviors in children with and without developmental regression. Journal of Developmental and Physical Disabilities. 2018;30(1):17-26.

30.	Ewen JB, Marvin AR, Law K, Lipkin PH. Epilepsy and autism severity: a study of 6,975 children. Autism Research. 2019;12(8):1251-9.

31.	Fang H, Wu Q, Li Y, Ren Y, Li C, Xiao X, et al. Structural networks in children with autism spectrum disorder with regression: a graph theory study. Behavioural Brain Research. 2020;378:112262.

32.	Fombonne E, Chakrabarti S. No evidence for a new variant of measles-mumps-rubella–induced autism. Pediatrics. 2001;108(4):e58-e.

33.	Frye RE, Cakir J, Rose S, Delhey L, Bennuri SC, Tippett M, et al. Early life metal exposure dysregulates cellular bioenergetics in children with regressive autism spectrum disorder. Transl Psychiatry. 2020;10(1):223.

34.	Frye RE, Rincon N, McCarty PJ, Brister D, Scheck AC, Rossignol DA. Biomarkers of mitochondrial dysfunction in autism spectrum disorder: A systematic review and meta-analysis. Neurobiology of Disease. 2024:106520.

35.	Furley K, Hunter MF, Fahey M, Williams K. Diagnostic findings and yield of investigations for children with developmental regression. Am J Med Genet, Part A. 2024;194(8):e63607.

36.	Gadow KD, Perlman G, Weber RJ. Parent-reported developmental regression in autism: epilepsy, IQ, schizophrenia spectrum symptoms, and special education. J Autism Dev Disord. 2017;47(4):918-26.

37.	Gagnon D, Zeribi A, Douard E, Courchesne V, Rodriguez-Herreros B, Huguet G, et al. Bayonet-shaped language development in autism with regression: a retrospective study. Molecular autism. 2021;12(1):35.

38.	Gaitanis J, Nie D, Hou T, Frye R. Developmental regression followed by epilepsy and aggression: a new syndrome in autism spectrum disorder? J Pers Med. 2023;13(7):1049.

39.	Gevezova M, Ivanov Z, Pacheva I, Timova E, Kazakova M, Kovacheva E, et al. Bioenergetic and Inflammatory Alterations in Regressed and Non-Regressed Patients with Autism Spectrum Disorder. Journal of Molecular Sciences. 2024;25(15):8211.

40.	Ghaziuddin M. Catatonia: a common cause of late regression in autism. Front Psychiatry. 2021;12:674009.

41.	Giannotti F, Cortesi F, Cerquiglini A, Miraglia D, Vagnoni C, Sebastiani T, et al. An investigation of sleep characteristics, EEG abnormalities and epilepsy in developmentally regressed and non-regressed children with autism. J Autism Dev Disord. 2008;38(10):1888-97.

42.	Giannotti F, Cortesi F, Cerquiglini A, Vagnoni C, Valente D. Sleep in children with autism with and without autistic regression. Journal of Sleep 2011;20(2):338-47.

43.	Goez H, Nielsen CC, Bryan S, Clark B, Zwaigenbaum L, Yamamoto SS, et al. Autistic regression and exposure to industrial chemicals: preliminary observations. Canadian Journal of Neurological Sciences. 2024;51(2):289-92.

44.	Goin-Kochel RP, Myers BJ. Congenital versus regressive onset of autism spectrum disorders: Parents' beliefs about causes. Focus on Autism and Other Developmental Disabilities. 2005;20(3):169-79.

45.	Goin-Kochel RP, Esler AN, Kanne SM, Hus V. Developmental regression among children with autism spectrum disorder: Onset, duration, and effects on functional outcomes. Research in Autism Spectrum Disorders. 2014;8(7):890-8.

46.	Goin-Kochel RP, Mire SS, Dempsey AG. Emergence of autism spectrum disorder in children from simplex families: relations to parental perceptions of etiology. J Autism Dev Disord. 2015;45(5):1451-63.

47.	Goin-Kochel RP, Mire SS, Dempsey AG, Fein RH, Guffey D, Minard CG, et al. Parental report of vaccine receipt in children with autism spectrum disorder: Do rates differ by pattern of ASD onset? Vaccine. 2016;34(11):1335-42.

48.	Goldberg WA, Osann K, Filipek PA, Laulhere T, Jarvis K, Modahl C, et al. Language and other regression: assessment and timing. J Autism Dev Disord. 2003;33(6):607-16.

49.	Goldberg WA, Thorsen KL, Osann K, Spence MA. Use of home videotapes to confirm parental reports of regression in autism. J Autism Dev Disord. 2008;38(6):1136-46.

50.	Hagberg B, Aicardi J, Dias K, Ramos O. A progressive syndrome of autism, dementia, ataxia, and loss of purposeful hand use in girls: Rett's syndrome: report of 35 cases. Annals of neurology. 1983;14(4):471-9.

51.	Hahn LJ, Brady NC, Warren SF, Fleming KK. Do children with fragile X syndrome show declines or plateaus in adaptive behavior? American Journal on Intellectual and Developmental Disabilities. 2015;120(5):412-32.

52.	Han L, Guan L, Zhang Z, Li W, Li J, Bao C, et al. Risk factors and clinical characteristics of autism spectrum disorder with regression in China. Autism Research. 2023;16(9):1836-46.

53.	Hansen RL, Ozonoff S, Krakowiak P, Angkustsiri K, Jones C, Deprey LJ, et al. Regression in autism: prevalence and associated factors in the CHARGE Study. Ambulatory Pediatrics. 2008;8(1):25-31.

54.	Havdahl A, Farmer C, Surén P, Øyen AS, Magnus P, Susser E, et al. Attainment and loss of early social‐communication skills across neurodevelopmental conditions in the Norwegian Mother, Father and Child Cohort Study. Journal of Child Psychology and Psychiatry. 2024;65(5):610-9.

55.	Havdahl A, Farmer C, Schjolberg S, Oyen A-S, Suren P, Reichborn-Kjennerud T, et al. Age of walking and intellectual ability in autism spectrum disorder and other neurodevelopmental disorders: a population-based study. Journal of child psychology and psychiatry, and allied disciplines. 2021;62(9):1070-8.

56.	Hrdlicka M, Komarek V, Propper L, Kulisek R, Zumrova A, Faladova L, et al. Not EEG abnormalities but epilepsy is associated with autistic regression and mental functioning in childhood autism. European child & adolescent psychiatry. 2004;13(4):209-13.

57.	Hu C, Yang F, Yang T, Chen J, Dai Y, Jia F, et al. A multi-center study on the relationship between developmental regression and disease severity in children with autism spectrum disorders. Frontiers in psychiatry. 2022;13:796554.

58.	Hu C, Yang T, Chen J, Dai Y, Wei H, Wu Q, et al. Phenotypic characteristics and rehabilitation effect of children with regressive autism spectrum disorder: a prospective cohort study. BMC Psychiatry. 2024;24(1):514.

59.	Jain R, Singh A, Mittal M, Talukdar B. Vitamin B12 deficiency in children: a treatable cause of neurodevelopmental delay. Journal of child neurology. 2015;30(5):641-3.

60.	Jakati P, Naskar S, Saha B, Chowdhury S. Developmental regression in children with autism based on parental reports: A cross-sectional study from eastern India. Indian Journal of Psychiatry. 2020;62:S126-S7.

61.	Jones LA, Campbell JM. Clinical characteristics associated with language regression for children with autism spectrum disorders. J Autism Dev Disord. 2010;40(1):54-62.

62.	Jyonouchi H, Sun S, Le H. Proinflammatory and regulatory cytokine production associated with innate and adaptive immune responses in children with autism spectrum disorders and developmental regression. J Neuroimmunol. 2001;120(1-2):170-9.

63.	Kaba D, Canlı MA. Exploration of risk factors for language regression according to parent reports in Turkish children with autism spectrum disorder. Psychiatry and Clinical Psychopharmacology. 2024;34(2):172.

64.	Kalb LG, Law J, Landa R, Law PA. Onset patterns prior to 36 months in autism spectrum disorders. J Autism Dev Disord. 2010;40(11):1389-402.

65.	Karimzadeh P, Jafari N, Biglari HN, Jabbehdari S, Zadeh SK, Abadi FA, et al. Neurometabolic diagnosis in children who referred as neurodevelopmental delay (a practical criteria, in Iranian pediatric patients). Iran J Child Neurol. 2016;10(3):73.

66.	Keene DL, Sutcliffe T, Harman P, Grenier D. Surveillance for progressive intellectual and neurological deterioration in the Canadian paediatric population. Journal of Neurological Sciences. 2004;31(2):220-4.

67.	Kehrer C, Blumenstock G, Gieselmann V, Kragelon-Mann I, Leukonet G. The natural course of gross motor deterioration in metachromatic leukodystrophy. Developmental Medicine & Child Neurology. 2011;53(9):850-5.

68.	Kehrer C, Groeschel S, Kustermann-Kuhn B, Bürger F, Köhler W, Kohlschütter A, et al. Language and cognition in children with metachromatic leukodystrophy: onset and natural course in a nationwide cohort. Orphanet Journal of Rare Diseases. 2014;9(1):18.

69.	Kern JK, Geier DA, Geier MR. Evaluation of regression in autism spectrum disorder based on parental reports. North American Journal of Medical Sciences. 2014;6(1):41.

70.	Kobayashi R, Murata T. Setback phenomenon in autism and longterm prognosis. Acta Psychiatrica Scandinavica. 1998;98(4):296-303.

71.	Kohlenberg TM, Trelles MP, McLarney B, Betancur C, Thurm A, Kolevzon A. Psychiatric illness and regression in individuals with Phelan-McDermid syndrome. J Neurodev Disord. 2020;12(1):7.

72.	Kumar S, Karmakar P, Mohanan A. Language regression in children with autism spectrum disorders. International Journal of Pediatric Otorhinolaryngology. 2014;78(2):334-8.

73.	Kurita H, Kita M, Miyake Y. A comparative study of development and symptoms among disintegrative psychosis and infantile autism with an without speech loss. J Autism Dev Disord. 1992;22(2):175-88.

74.	Kurita H. Specificity and developmental consequences of speech loss in children with pervasive developmental disorders. Psychiatry and Clinical Neurosciences. 1996;50(4):181-4.

75.	Kurita H, Koyama T, Setoya Y, Shimizu K, Osada H. Validity of childhood disintegrative disorder apart from autistic disorder with speech loss. European Child & Adolescent Psychiatry. 2004;13(4):221-6.

76.	Lainhart JE, Ozonoff S, Coon H, Krasny L, Dinh E, Nice J, et al. Autism, regression, and the broader autism phenotype. Am J Med Genet. 2002;113(3):231-7.

77.	Lance EI, York JM, Lee L-C, Zimmerman AW. Association between regression and self injury among children with autism. Research in Developmental Disabilities. 2014;35(2):408-13.

78.	Lee J, Leonard H, Piek J, Downs J. Early development and regression in Rett syndrome. Clinical Genetics. 2013;84(6):572-6.

79.	Li X, Zhou P, Li Q, Peng B, Cun Y, Dai Y, et al. Regressive autism spectrum disorder: High levels of total secreted amyloid precursor protein and secreted amyloid precursor protein-α in plasma. Front Psychiatry. 2022;13:809543.

80.	Lord C, Shulman C, DiLavore P. Regression and word loss in autistic spectrum disorders. Journal of Child Psychology and Psychiatry. 2004;45(5):936-55.

81.	Luyster R, Richler J, Risi S, Hsu W-L, Dawson G, Bernier R, et al. Early regression in social communication in autism spectrum disorders: A CPEA study. Developmental Neuropsychology. 2005;27(3):311-36.

82.	Maestro S, Muratori F, Cesari A, Pecini C, Apicella F, Stern D. A view to regressive autism through home movies. Is early development really normal? Acta Psychiatrica Scandinavica. 2006;113(1):68-72.

83.	Malek M, Ashraf-Ganjouei A, Moradi K, Bagheri S, Mohammadi M-R, Akhondzadeh S. Prednisolone as Adjunctive Treatment to Risperidone in Children With Regressive Type of Autism Spectrum Disorder: A Randomized, Placebo-Controlled Trial. Clinical neuropharmacology. 2020;43(2):39-45.

84.	Malhi P, Singhi P. Regression in children with autism spectrum disorders. The Indian Journal of Pediatrics. 2012;79(10):1333-7.

85.	Manelis L, Meiri G, Ilan M, Flusser H, Michaelovski A, Faroy M, et al. Language regression is associated with faster early motor development in children with autism spectrum disorder. Autism Research. 2020;13(1):145-56.

86.	Marafi D, Suter B, Schultz R, Glaze D, Pavlik VN, Goldman AM. Spectrum and time course of epilepsy and the associated cognitive decline in MECP2 duplication syndrome. Neurology. 2019;92(2):e108-e14.

87.	Martin-Borreguero P, Gómez-Fernández AR, De La Torre-Aguilar MJ, Gil-Campos M, Flores-Rojas K, Perez-Navero JL. Children with autism spectrum disorder and neurodevelopmental regression present a severe pattern after a follow-up at 24 months. Front Psychiatry. 2021;12:644324.

88.	Matson JL, Wilkins J, Fodstad JC. Children with autism spectrum disorders: A comparison of those who regress vs. those who do not. Developmental Neurorehabilitation. 2010;13(1):37-45.

89.	McVicar KA, Ballaban-Gil K, Rapin I, Moshé SL, Shinnar S. Epileptiform EEG abnormalities in children with language regression. Neurology. 2005;65(1):129-31.

90.	Meilleur AA, Fombonne E. Regression of language and non‐language skills in pervasive developmental disorders. JIDR. 2009;53(2):115-24.

91.	Mire SS, Anderson JR, Manis JK, Hughes K, Raff NS, Goin-Kochel RP. Using teacher ratings to investigate developmental regression as a potential indicator of school-age symptoms in students with autism spectrum disorder. School Mental Health. 2018;10(1):77-90.

92.	Molloy CA, Morrow AL, Meinzen-Derr J, Dawson G, Bernier R, Dunn M, et al. Familial Autoimmune Thyroid Disease as a Risk Factor for Regression in Children with Autism Spectrum Disorder: A CPEA Study: Molloy et al. J Autism Dev Disord. 2006;36(3):317-24.

93.	Nickel M, Simonati A, Jacoby D, Lezius S, Kilian D, Van de Graaf B, et al. Disease characteristics and progression in patients with late-infantile neuronal ceroid lipofuscinosis type 2 (CLN2) disease: an observational cohort study. The Lancet Child & Adolescent Health. 2018;2(8):582-90.

94.	Nordahl CW, Lange N, Li DD, Barnett LA, Lee A, Buonocore MH, et al. Brain enlargement is associated with regression in preschool-age boys with autism spectrum disorders. Proceedings of the National Academy of Sciences. 2011;108(50):20195-200.

95.	Nunn K, Williams K, Ouvrier R. The Australian childhood dementia study. European child & adolescent psychiatry. 2002;11(2):63-70.

96.	Ošlejšková H, Dusek L, Makovska A, Pejcochova J, Autrata R, Slapak I. Complicated relationship between autism with regression and epilepsy. Neuroendocrinol Lett. 2008;29(4):558-70.

97.	Ozonoff S, Williams BJ, Landa R. Parental report of the early development of children with regressive autism: the delays-plus-regression phenotype. Autism. 2005;9(5):461-86.

98.	Ozonoff S, Young GS, Goldring S, Greiss-Hess L, Herrera AM, Steele J, et al. Gross motor development, movement abnormalities, and early identification of autism. J Autism Dev Disord. 2008;38(4):644-56.

99.	Ozonoff S, Iosif A-M, Young GS, Hepburn S, Thompson M, Colombi C, et al. Onset patterns in autism: correspondence between home video and parent report. Journal of the American Academy of Child & Adolescent Psychiatry. 2011;50(8):796-806.

100.	Ozonoff S, Gangi D, Hanzel EP, Hill A, Hill MM, Miller M, et al. Onset patterns in autism: Variation across informants, methods, and timing. Autism Research. 2018;11(5):788-97.

101.	Pardo CA, Buckley A, Thurm A, Lee L-C, Azhagiri A, Neville DM, et al. A pilot open-label trial of minocycline in patients with autism and regressive features. J Neurodev Disord. 2013;5(1):9.

102.	Parr JR, Le Couteur A, Baird G, Rutter M, Pickles A, Fombonne E, et al. Early developmental regression in autism spectrum disorder: evidence from an international multiplex sample. J Autism Dev Disord. 2011;41(3):332-40.

103.	Pérez‐Poyato MS, Milà Recansens M, Ferrer Abizanda I, Montero Sánchez R, Rodríguez‐Revenga L, Cusí Sánchez V, et al. Juvenile neuronal ceroid lipofuscinosis: clinical course and genetic studies in Spanish patients. Journal of Inherited Metabolic Disease. 2011;34(5):1083-93.

104.	Pérez-Poyato MS, Marfa MP, Abizanda IF, Rodriguez-Revenga L, Sánchez VC, González MJM, et al. Late infantile neuronal ceroid lipofuscinosis: mutations in the CLN2 gene and clinical course in Spanish patients. Journal of Child Neurology. 2013;28(4):470-8.

105.	Peters S, Hundley R, Wilson A, Carvalho C, Lupski J, Ramocki M. Brief report: regression timing and associated features in *MECP2* duplication syndrome. J Autism Dev Disord. 2013;43(10):2484-90.

106.	Pickles A, Simonoff E, Conti‐Ramsden G, Falcaro M, Simkin Z, Charman T, et al. Loss of language in early development of autism and specific language impairment. Journal of Child Psychology and Psychiatry. 2009;50(7):843-52.

107.	Plaza-Díaz J, Gómez-Fernández A, Chueca N, Torre-Aguilar MJdl, Gil Á, Perez-Navero JL, et al. Autism spectrum disorder (ASD) with and without mental regression is associated with changes in the fecal microbiota. Nutrients. 2019;11(2):337.

108.	Prasad A, Stefanelli M, Nagarajan L. Seizure exacerbation and developmental regression with carbamazepine. Canadian Journal of Neurological Sciences. 1998;25(4):287-94.

109.	Prescott KE, Ellis Weismer S. Children with ASD and communication regression: Examining pre-loss skills and later language outcomes through the preschool years. J Autism Dev Disord. 2022;52(5):1956-70.

110.	Priyanka C, Christopher R, Nagappa M, Kommu JVS, Krishnadevaraje MB, Gunasekaran DM, et al. Targeted metabolomics in children with autism spectrum disorder with and without developmental regression. Metabolic Brain Disease. 2025;40(4):184.

111.	Prosperi M, Guiducci L, Peroni DG, Narducci C, Gaggini M, Calderoni S, et al. Inflammatory Biomarkers are Correlated with Some Forms of Regressive Autism Spectrum Disorder. Brain Sci. 2019;9(12).

112.	Rangel-Huerta O, Gomez-Fernández A, De La Torre-Aguilar M, Gil A, Perez-Navero J, Flores-Rojas K, et al. Metabolic profiling in children with autism spectrum disorder with and without mental regression: Preliminary results from a cross-sectional case–control study. Metabolomics. 2019;15(7):99.

113.	Reyes N, Soke GN, Wiggins L, Barger B, Moody E, Rosenberg C, et al. Social and language regression: characteristics of children with autism spectrum disorder in a community-based sample. Journal of developmental and physical disabilities. 2024;36(4):713-28.

114.	Richler J, Luyster R, Risi S, Wan-Ling H, Dawson G, Bernier R, et al. Is there a'regressive phenotype'of Autism Spectrum Disorder associated with the measles-mumps-rubella vaccine? A CPEA Study. J Autism Dev Disord. 2006;36(3):299.

115.	Romani M, Kraoua I, Micalizzi A, Klaa H, Benrhouma H, Drissi C, et al. Infantile and childhood onset PLA 2G6‐associated neurodegeneration in a large North African cohort. Eur J Neurol. 2015;22(1):178-86.

116.	Sandler RH, Finegold SM, Bolte ER, Buchanan CP, Maxwell AP, Väisänen M-L, et al. Short-term benefit from oral vancomycin treatment of regressive-onset autism. Journal of child neurology. 2000;15(7):429-35.

117.	Santoro JD, Partridge R, Tanna R, Pagarkar D, Khoshnood M, Rehmani M, et al. Evidence of neuroinflammation and immunotherapy responsiveness in individuals with down syndrome regression disorder. J Neurodev Disord. 2022;14(1):35.

118.	Saraf UU, Asranna A, Menon RN, Mohan P M, Vp V, Radhakrishnan A, et al. Predictors of one-year language and seizure outcomes in children with epileptic encephalopathy with continuous spike-and-wave during sleep (CSWS). Seizure. 2020;81:315-24.

119.	Scott O, Shi D, Andriashek D, Clark B, Goez HR. Clinical clues for autoimmunity and neuroinflammation in patients with autistic regression. Dev Med Child Neurol. 2017;59(9):947-51.

120.	Seegmüller C, Deonna T, Mayor Dubois C, Valenti‐Hirsch MP, Hirsch E, Metz‐Lutz MN, et al. Long‐term outcome after cognitive and behavioral regression in nonlesional epilepsy with continuous spike‐waves during slow‐wave sleep. Epilepsia. 2012;53(6):1067-76.

121.	Shapiro EG, Nestrasil I, Delaney KA, Rudser K, Kovac V, Nair N, et al. A Prospective Natural History Study of Mucopolysaccharidosis Type IIIA. J Pediatr. 2016;170:278-87.e1-4.

122.	Sheth J, Mistri M, Bhavsar R, Sheth F, Kamate M, Shah H, et al. Lysosomal Storage Disorders in Indian Children with Neuroregression Attending a Genetic Center. Indian Pediatr. 2015;52(12):1029-33.

123.	Shinnar S, Rapin I, Arnold S, Tuchman RF, Shulman L, Ballaban-Gil K, et al. Language regression in childhood. Pediatr Neurol. 2001;24(3):185-91.

124.	Shoffner J, Hyams L, Langley GN, Cossette S, Mylacraine L, Dale J, et al. Fever plus Mitochondrial disease could be risk factors for autistic regression. J Child Neurol. 2010;25(4):429-34.

125.	Shumway S, Thurm A, Swedo SE, Deprey L, Barnett LA, Amaral DG, et al. Brief report: symptom onset patterns and functional outcomes in young children with autism spectrum disorders. J Autism Dev Disord. 2011;41(12):1727-32.

126.	Singh K, Singh IN, Diggins E, Connors SL, Karim MA, Lee D, et al. Developmental regression and mitochondrial function in children with autism. Ann Clin Transl Neurol. 2020;7(5):683-94.

127.	Siperstein R, Volkmar F. Brief report: Parental reporting of regression in children with pervasive developmental disorders. J Autism Dev Disord. 2004;34(6):731-4.

128.	Stellitano LA, Winstone AM, Van der Knaap MS, Verity CM. Leukodystrophies and genetic leukoencephalopathies in childhood: a national epidemiological study. Developmental Medicine & Child Neurology. 2016;58(7):680-9.

129.	Tamanaha AC, Machado GM, Loebmann C, Perissinoto J. Process of speech acquisition and development of autistic children with or without autistic regression. Codas. 2014;26(4):265-9.

130.	Tamouza R, Fernell E, Eriksson MA, Anderlid BM, Manier C, Mariaselvam CM, et al. HLA Polymorphism in Regressive and Non-Regressive Autism: A Preliminary Study. Autism Research. 2020;13(2):182-6.

131.	Taylor B, Miller E, Lingam R, Andrews N, Simmons A, Stowe J. Measles, mumps, and rubella vaccination and bowel problems or developmental regression in children with autism: population study. Br Med J. 2002;324(7334):393-6.

132.	Thompson L, Gillberg C, Landberg S, Kantzer AK, Miniscalco C, Barnevik Olsson M, et al. Autism with and without regression: a two-year prospective longitudinal study in two population-derived Swedish cohorts. J Autism Dev Disord. 2019;49(6):2281-90.

133.	Thurm A, Manwaring SS, Luckenbaugh DA, Lord C, Swedo SE. Patterns of skill attainment and loss in young children with autism. Dev Psychopathol. 2014;26(1):203-14.

134.	Tinker RJ, Falk MJ, Goldstein A, George-Sankoh I, Xiao R, Adang L, et al. Early developmental delay in Leigh syndrome spectrum disorders is associated with poor clinical prognosis. Mol Genet Metab. 2022;135(4):342-9.

135.	Tonekaboni SH, Iaboni A, Trost B, Reuter M, Lindenmaier Z, Kushki A, et al. Comparative analysis of phenotypic and genotypic differences between individuals affected by regressive and non‐regressive autism: a cross‐sectional study. Autism Research. 2025;18(6):1290-300.

136.	Torrente F, Anthony A, Heuschkel RB, Thomson MA, Ashwood P, Murch SH. Focal-enhanced gastritis in regressive autism with features distinct from Crohn's and Helicobacter pylori gastritis. Am J Gastroenterol. 2004;99(4):598-605.

137.	Tuchman RF, Rapin I. Regression in pervasive developmental disorders: seizures and epileptiform electroencephalogram correlates. Pediatrics. 1997;99(4):560-6.

138.	Uchiyama T, Kurosawa M, Inaba Y. MMR-vaccine and regression in autism spectrum disorders: negative results presented from Japan. J Autism Dev Disord. 2007;37(2):210-7.

139.	Urbanowicz A, Downs J, Girdler S, Ciccone N, Leonard H. Aspects of speech‐language abilities are influenced by *MECP2* mutation type in girls with Rett syndrome. Am J Med Genet, Part A. 2015;167(2):354-62.

140.	Valicenti-McDermott MD, McVicar K, Cohen HJ, Wershil BK, Shinnar S. Gastrointestinal symptoms in children with an autism spectrum disorder and language regression. Pediatric Neurology. 2008;39(6):392-8.

141.	van Iterson L, Zijlstra BJ, Augustijn PB, van der Leij A, de Jong PF. Duration of epilepsy and cognitive development in children: A longitudinal study. Neuropsychology. 2014;28(2):212.

142.	Verity CM, Winstone AM, Stellitano L, Krishnakumar D, Will R, McFarland R. The clinical presentation of mitochondrial diseases in children with progressive intellectual and neurological deterioration: a national, prospective, population‐based study. Developmental Medicine & Child Neurology. 2010;52(5):434-40.

143.	Verity C, Winstone AM, Stellitano L, Will R, Nicoll A. The epidemiology of progressive intellectual and neurological deterioration in childhood. Arch Dis Child. 2010;95(5):361-4.

144.	Verity C, Baker E, Maunder P, Pal S, Winstone A. Differential diagnosis of progressive intellectual and neurological deterioration in children. Developmental Medicine & Child Neurology. 2021;63(3):287-94.

145.	Wasilewska J, Kaczmarski M, Stasiak-Barmuta A, Tobolczyk J, Kowalewska E. Low serum IgA and increased expression of CD23 on B lymphocytes in peripheral blood in children with regressive autism aged 3-6 years old. Arch Med Sci. 2012;8(2):324-31.

146.	Webb SJ, Nalty T, Munson J, Brock C, Abbott R, Dawson G. Rate of head circumference growth as a function of autism diagnosis and history of autistic regression. J Child Neurol. 2007;22(10):1182-90.

147.	Werner E, Dawson G. Validation of the phenomenon of autistic regression using home videotapes. Arch Gen Psychiatry. 2005;62(8):889-95.

148.	Wiggins LD, Rice CE, Baio J. Developmental regression in children with an autism spectrum disorder identified by a population-based surveillance system. Autism. 2009;13(4):357-74.

149.	Wilson S, Djukic A, Shinnar S, Dharmani C, Rapin I. Clinical characteristics of language regression in children. Dev Med Child Neurol. 2003;45(8):508-14.

150.	Woo EJ, Ball R, Landa R, Zimmerman AW, Braun MM. Developmental regression and autism reported to the Vaccine Adverse Event Reporting System. Autism. 2007;11(4):301-10.

151.	Worley G, Crissman BG, Cadogan E, Milleson C, Adkins DW, Kishnani PS. Down syndrome disintegrative disorder: New-onset autistic regression, dementia, and insomnia in older children and adolescents with Down syndrome. J Child Neurol 2015;30(9):1147-52.

152.	Yan J, Han VX, Jones HF, Couttas TA, Jieu B, Leweke FM, et al. Cerebrospinal fluid metabolomics in autistic regression reveals dysregulation of sphingolipids and decreased β-hydroxybutyrate. EBioMedicine. 2025;114:105664.

153.	Yin J, Chun C-A, Zavadenko NN, Pechatnikova NL, Naumova OY, Doddapaneni HV, et al. Next generation sequencing of 134 children with autism spectrum disorder and regression. Genes. 2020;11(8):853.

154.	Yurumez E, Cikili-Uytun M, Kaymak B, Dogan O, Ozturk H, Baysar-Kanoglu B, et al. Neurodegeneration in Autism: a study of clusterin, very long-chain fatty acids, and carnitine. J Mol Neurosci. 2025;75(1):18.

155.	Zappella M. Autistic regression with and without EEG abnormalities followed by favourable outcome. Brain Dev. 2010;32(9):739-45.

156.	Zhang Y, Xu Q, Liu J, Li SC, Xu X. Risk factors for autistic regression: results of an ambispective cohort study. J Child Neurol. 2012;27(8):975-81.

157.	Zhang L, Li K, Zhang C, Qi X, Zheng N, Wang G. Arcuate Fasciculus in Autism Spectrum Disorder Toddlers with Language Regression. Open Medicine. 2018;13(1):90-5.
